# Azidophosphonium salt-directed chemoselective synthesis of (*E*)/(*Z*)-cinnamyl-1*H*-triazoles and regiospecific access to bromomethylcoumarins from Morita–Baylis–Hillman adducts

**DOI:** 10.3762/bjoc.16.130

**Published:** 2020-07-01

**Authors:** Soundararajan Karthikeyan, Radha Krishnan Shobana, Kamarajapurathu Raju Subimol, J Helen Ratna Monica, Ayyanoth Karthik Krishna Kumar

**Affiliations:** 1 Organic & Material Chemistry Research Laboratory, Department of Chemistry, The American College, Madurai, Tamil Nadu, India; 2 Department of Chemistry, Fatima College, Madurai, Tamil Nadu, India

**Keywords:** halomethylcoumarin, Morita–Baylis–Hillman adducts, organocatalyst, phosphonium salt, triazolation

## Abstract

The direct transformation of Morita–Baylis–Hillman (MBH) adducts into molecules of interest is a crucial process wherein allylic hydroxy-protected or halogenated MBH adducts are commonly preferred. Herein, we report an azidophosphonium salt (AzPS)-catalysed straight forward protocol for synthesising structurally demanding (*E*)/(*Z*)-cinnamyl-1*H*-1,2,3-triazoles and halomethylcoumarins from MBH adducts. The novel methodology, efficient catalyst, and direct utilization of MBH adducts under mild reaction conditions qualify the reported procedures as powerful synthetic tools.

## Introduction

The presence of versatile functional groups in close proximity classifies Morita–Baylis–Hillman adducts as privileged key scaffolds for synthetic organic chemists. Accordingly, MBH adducts have been explored as strategic intermediates for the synthesis of interesting molecules, such as carbamates of unsaturated β-amino acids [1], β-phenylsylfenyl-α-cyanohydrocinnamaldhydes [2], 2-alkylcarbonyl-1-indanols [3], dihydropyrazoles [4], tetrahydroacridines [5], γ-lactams [6], quinolin-5-ones [7], spirobisglutarimides [8], indolizines [9], and spiro carbocyclic frameworks [10]. However, most of the reported synthetic transformations utilize either allylic hydroxy-protected or allyl halide-substituted MBH adducts [11–23].

**Scheme 1:**
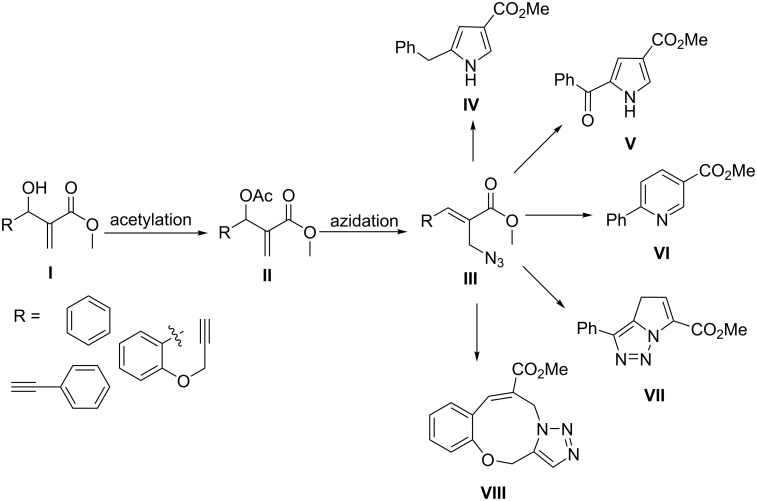
Literature-reported cycloaddition reactions of MBH acetates involving azides and alkynes [24–28].

Among the known synthetic transformations using functionalized MBH adducts, cycloaddition reactions are challenging and attractive for synthetic organic chemists. In this context, acetate-functionalized Morita–Baylis–Hillman adducts have been extensively utilized over other precursors. For example, heterocycles such as, pyrroles (e.g., **IV**) [24], keto pyrroles (e.g., **V**) [25], pyridines (e.g., **VI**) [26], pyrrolotriazoles (e.g., **VII**) [27], and triazolobenzoxazonines (e.g., **VIII**) [28] result from MBH acetates (Scheme 1). From these synthetic elaborations, three successive steps are universally utilized: (i) acetylation, (ii) azidation, and (iii) cycloaddition to produce **IV**–**VIII**. In spite of the broad scope and synthetic utility, it is evident that the multistep synthetic methodology is the only existing module for cycloaddition reactions.

**Scheme 2:**
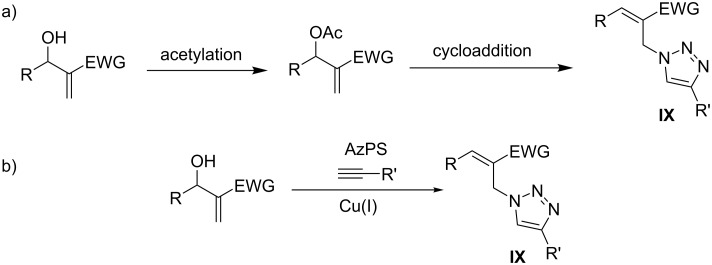
Synthetic methodologies for triazolations of MBH adducts. a) Literature-reported indirect triazolation of MBH adducts [32–33]. b) This work: phosphonium salt-catalysed triazolation of MBH adducts.

Our research group is focused on developing one-pot synthetic transformations for complex molecules [29–31]. Two individual research groups have reported the multistep pathway to access the cinnamyl-1*H*-1,2,3-triazole derivatives **IX** from acetates of MBH adducts (Scheme 2) [32–33]. The other preferable moiety for triazole transformations is the allyl halide of MBH adducts, however, the vicinity of its (*E*)- and (*Z*)-isomers restricts their use as a favourable starting moiety [34]. After a careful bibliographic investigation, it became evident that there were no one-pot protocols for direct transformations of MBH adducts to cinnamyl triazoles. The outcome of developing a one-pot synthetic strategy will be worthwhile for pharmacologically important triazoles, such as isavuconazole, tazobactam, and ravuconazole [35].

## Results and Discussion

Initially, phosphonium salts were barely utilised or exploited in synthetic transformations. Later, in 2014, several organic transformations employed quaternary phosphonium salts as favourable catalysts [36]. Their synthetic utility was not only confined to catalysis, but they were also used as intermediates for the synthesis of 1*H*-indazoles [37], as promoters for stereoselective rearrangements [38], and as temporary protectors of O,P-acetals [39], which branded them as promising motifs. The above reports and the Lewis acid character of quaternary phosphonium salts (QPS) [40–48] qualifies them as reliable catalysts for the proposed methodology. The most elaborate process in the proposed methodology is the protection and elimination of the allylic hydroxy group. We believe that this crucial strategy could be primarily resolved by a quaternary phosphonium salt. After the initial screening of various quaternary phosphonium salts, the azidophosphonium salt [Ph_3_P^+^CBr_3_]N_3_^−^, reported by Blanco and co-workers, was opted to accomplish our goal [49–51]. The AzPS surprisingly synchronised with the functional and structural requirements of the proposed work. The azidophosphonium salt was generated and purified according to a modified literature procedure [49].

The one-pot model reaction was investigated using the MBH adduct **1a** (1 equiv) and propargyl alcohol (**2a**, 1.2 equiv) in presence of the AzPS [Ph_3_P^+^CBr_3_]N_3_^−^ (see Supporting Information File 1 for the substituent patterns of the compounds **1a**–**o**). In this precedent reaction, the adduct **1a** and propargyl alcohol (**2a**) in THF were treated with the AzPS (1 equiv) and CuI (3 mol %) at room temperature. To our expectations, the reaction afforded the (*E*)-cinnamyl-1*H*-1,2,3-triazole in a low yield of 24% (Table 1, entry 1). Thereby, we anticipated that an increase in the proportion of the AzPS would substantially increase the yield of **3a** (Table 1, entries 2 and 3), but unexpectedly, the reaction demonstrated an unsatisfactory yield. Thereafter, on attempting the reaction with an improved ratio of CuI (5 mol %) and AzPS (2 equiv), the expected product **3a** was obtained in a moderate yield (71%, Table 1, entry 4). However, a further increase in the AzPS ascertained a gradual decrease in the yield of **3a** (Table 1, entries 5 and 6). The outcome of this analysis might have been due to the formation of large amounts of the byproduct triphenylphosphine oxide, which impeded the purification process and decreased the yield of **3a**. Alternative Cu(I) catalysts, CuCl and CuBr, were also used at 5 mol % with the AzPS (2 equiv), however, the combination showed no potential increase in the yield of **3a** (Table 1, entries 7 and 8). Comprehensive investigations on the proposed methodology revealed 2 equiv of the AzPS and 5 mol % of CuI as the optimized catalytic combination. Further, the optimized reaction was screened in presence of various solvents (Table 1, entries 9–13), and the outcome revealed acetonitrile as the most preferable solvent, yielding **3a** in 83% yield (Table 1, entry 11). Interestingly, the dilution of the reaction mixture did not alter the efficiency of this reaction.

**Table 1:** Optimization of the triazolation of the MBH adduct **1a**.

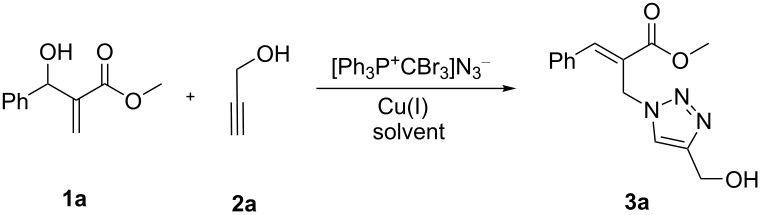				

entry	equiv of AzPS	Cu(I) salt (mol %)	solvent	yield (%)

1	1	CuI (3)	THF	24
2	1.5	CuI (3)	THF	33
3	2	CuI (3)	THF	42
4	2	CuI (5)	THF	71
5	3	CuI (5)	THF	45
6	4	CuI (5)	THF	36
7	2	CuCl (5)	THF	47
8	2	CuBr (5)	THF	54
9	2	CuI (5)	EtOAc	66
10	2	CuI (5)	acetone	69
11	2	CuI (5)	CH_3_CN	83
12	2	CuI (5)	DMF	64
13	2	CuI (5)	DMSO	69

**Scheme 3:**
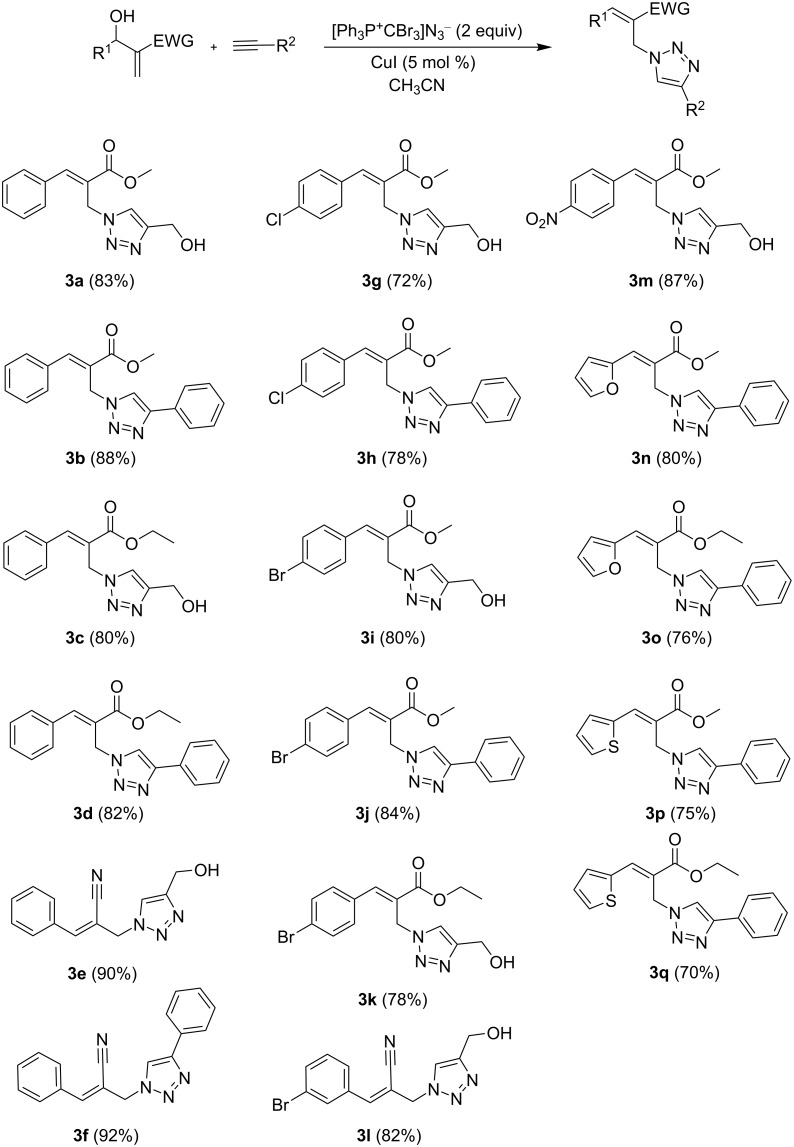
Scope of the one-pot cascade reaction of the unprotected Morita–Baylis–Hillman adducts **3a**–**q**.

The substrate scope of the optimized reaction and its limitations were further extended to structurally distinct MBH adducts (Scheme 3). The MBH adducts derived from methoxy and ethoxy acrylate stereochemically afforded the (*E*)-cinnamyl-1,4-disubstituted 1,2,3-triazole derivatives **3a**–**d**/**g**–**k/m**–**q** in a yield of 70–88%. Distinctively, the cyano acrylate-substituted MBH adduct stereoselectively afforded the (*Z*)-cinnamyl-1,4-disubstituted 1,2,3-triazole derivatives **3e**/**f**/**l** in a yield of 82–92%. Irrespective of the acetylene moiety, the MBH adducts derived from acrylonitrile comparatively afforded cinnamyl-1,4-disubstituted 1,2,3-triazoles at an improved yield compared to that of the methyl and ethyl counterparts. Notably, the MBH adducts derived from the *para*-bromo-, *para*-chloro-, and *para*-nitrobenzaldehydes favourably assisted the formation of the corresponding (*E*)-cinnamyl-1,4-disubstituted 1,2,3-triazole derivatives **3g**–**m** in a yield of 72–87%. Alternatively, the *ortho*- and *meta-*substituted aryl-MBH adducts were incompatible with the optimized reaction conditions, and this was presumably due to the apparent steric hindrance. Similarly, the MBH adducts derived from aliphatic aldehydes, salicylaldehydes, and methyl- or methoxy-substituted benzaldehydes were also inert under the optimized reaction conditions. Therefore, it is evident that the electronic variation of the substituents on the aromatic moiety of the MBH adducts played a crucial role in determining the outcome of the optimized reactions. We further extended the scope of this transformation to five-membered heterocyclic MBH adducts. To our delight, except pyrroles, the proposed methodology was amenable to MBH adducts of furan and thiophene (**3n**–**q**, 70–80%).

**Figure 1:**
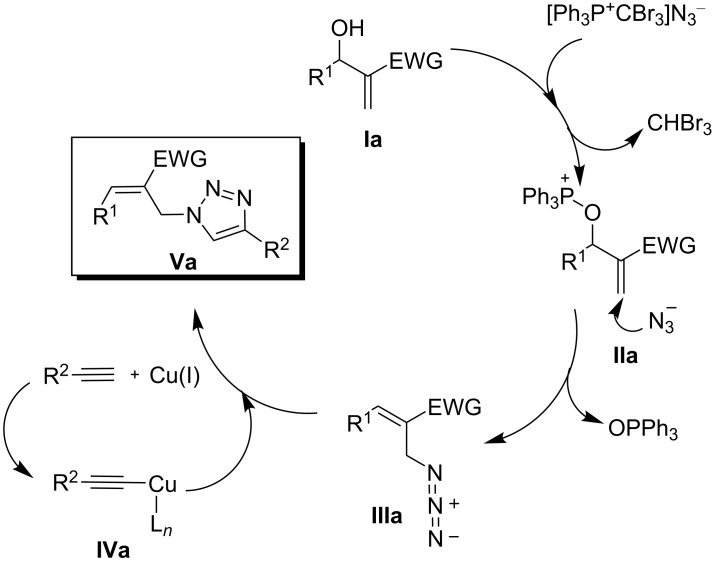
Proposed mechanism for the synthesis of 1,4-disubstituted triazoles.

The mechanistic pathway for the triazolation proceeded via a nucleophilic attack on the AzPS by the allylic alcohol of the MBH adduct **Ia**. Subsequently, the azide ion undergoes a nucleophilic attack on the allylic carbon atom of the oxyphosphonium intermediate **IIa** and generates the 2-azidoalkene **IIIa**. Interestingly, the consecutive nucleophilic attack by the azido ion smoothly initiates the allylic rearrangement and thereby facilitates the removal of the crucial phosphonium oxide. The outcome of this process is the structurally relevant azido moiety **IIIa**, which then undergoes a 1,3-dipolar cycloaddition with the copper acetylide **IVa** to furnish the 1,4-disubstituted 1,2,3-triazoles **Va** (Figure 1).

**Scheme 4:**
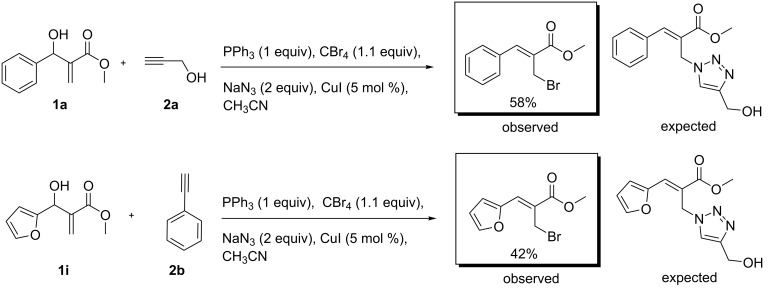
Comparative analysis of the sequential one-pot reaction.

At this stage, we sought to analyse the outcome of the proposed reaction following a sequential addition of the reagents utilised for the synthesis of AzPS. Therefore, a preliminary investigation was attempted using the MBH adduct **1a** (1 equiv) and propargyl alcohol (**2a**,1.2 equiv) in the presence of CuI (5 mol %), triphenylphosphine (1 equiv), bromomethane (1.1 equiv), and sodium azide (2 equiv). Unexpectedly, the reaction yielded (*Z*)-methyl-2-(bromomethyl)-3-phenylacrylate (58%) over the expected triazole. Similarly, the MBH adduct derived from furan, **1i**, and phenylacetylene (**2b**) also yielded (*Z*)-methyl 2-(bromomethyl)-3-(furan-2-yl)acrylate (42%) rather than the expected triazole (Scheme 4). Thereby, it was clearly evident that the addition of the individual reagents prevented the formation of complicated triazoles.

Interestingly, the MBH adducts derived from salicylaldehydes were inert to triazolations, surprisingly affords bromomethylcoumarin in the presence of AzPS and HBr. The reaction was optimized using salicylaldehyde (1 equiv) in the presence of AzPS (2 equiv) and HBr (2.0 equiv). The reaction afforded 6-(bromomethyl)coumarin (**4a**) in a yield of 78% (Table 2, entry 3). The synthetic utility of the reaction was further extended to *ortho*-vanillin and *para*-bromobenzaldehyde to afford the corresponding halomethylcoumarins (**4b**/**c**). However, this regiospecific transformation was restricted only to the MBH adducts derived from salicyladehydes and *tert*-butyl acrylate [52–53]. Among the reported methodologies on synthesis of halomethylcoumarins [54–55], the present methodology was attractive due to its good yield and the simple reaction conditions.

**Table 2:** Optimization of the reaction conditions for 3-(bromomethyl)coumarins from MBH adducts.

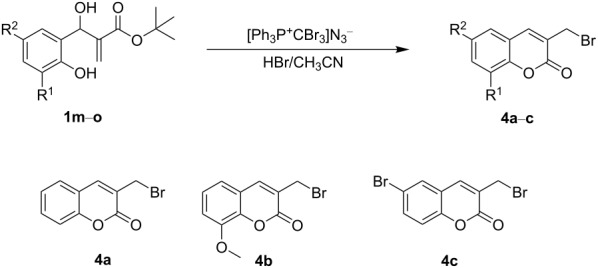				

entry	equiv of AzPS	equiv of HBr	solvent	yield **4a** (%)

1	2	–	CH_3_CN	–
2	2	1	CH_3_CN	33
3	2	2	CH_3_CN	78
4	2	3	CH_3_CN	62
5	2	4	CH_3_CN	31
6	3	2	CH_3_CN	65
7	4	2	CH_3_CN	57

**Figure 2:**
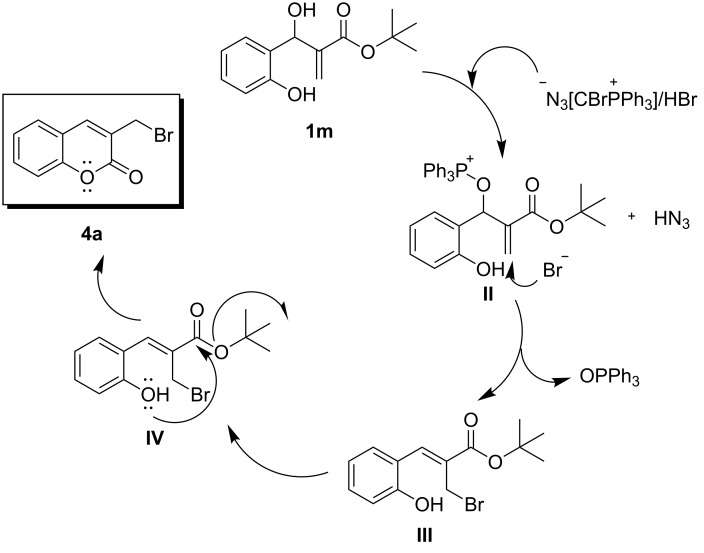
Proposed mechanism for the synthesis of 3-(bromomethyl)coumarins.

As shown in Figure 2, the mechanistic pathway for **4a**–**c** progressed via treating the MBH adduct (**1m**) with AzPS and HBr. The outcome of this process was the phosphonium-protected MBH moiety **Ib** and hydrazoic acid. The counter ion bromine facilitated the nucleophilic attack at the vinylic centre of **Ib** and the spontaneous removal of triphenylphosphine oxide to yield **IIb**. A consecutive intramolecular nucleophilic attack of the hydroxy moiety at the carbonyl carbon of **IIIb** further drove the cyclisation to afford the bromomethylcoumarin **4a**.

## Conclusion

In summary, we reported the first protocol on the quaternary phosphonium salt-mediated direct synthesis of cinnamyltriazoles and 3-(bromomethyl)coumarins from Morita–Baylis–Hillman adducts. In contrast to the contending reports on the synthesis of 1,2,3-triazoles and halomethylcoumarins from MBH adducts, our studies report moderate reaction conditions with an improved yield. The above investigation provides a useful synthetic tool for synthetic organic chemists. The synthesis of biologically important triazoles using the reported methodology is underway in our laboratory.

## Experimental

### General information

Chemicals were purchased from Sigma-Aldrich, Spectrochem (P) Ltd., Central Drug House (P) Ltd., and Rankem, India. All chemicals were used without further purification. The solvents were purified using standard procedures. ^1^H and ^13^C NMR spectra were recorded on a Bruker Avance 300 MHz spectrometer using CDCl_3_ and DMSO-*d*_6_ as the solvent. Tetramethylsilane (TMS) was used as an internal standard. Chemical shifts are given in δ relative to TMS. High-resolution mass spectra were recorded on an Agilent Technologies 6540 UHD accurate mass Q-TOF LC–MS spectrometer. Melting points are uncorrected. The compounds were purified using column chromatography on silica gel (100–200 mesh) using hexane/ethyl acetate and chloroform/methanol as eluent.

### Typical procedure for quaternary phosphonium salts

As described in [49]. Typically, triphenylphosphine, bromomethane, and sodium azide at a molar ratio of 1.1:1.1:5 were utilized for synthesising the quaternary phosphonium salt. Initially, triphenylphosphine and sodium azide were stirred at 0 °C in dimethylformamide (5 mL) for 30 minutes. To the mixture, bromomethane in DMF was added slowly to avoid a sudden increase in temperature. The reaction was slowly warmed to room temperature and stirred for another 30 minutes. Finally, the reaction was quenched by the addition of diethyl ether. The filtration of insoluble inorganic salts resulted in a transparent liquid, which, upon concentration by evaporation, provided a crude oily residue. The residue was dissolved in ethyl acetate, washed with brine, and dried over sodium sulphate to yield a clear oily residue of the quaternary phosphonium salt.

### Typical procedure for **1a–o**

As described in [52]. A mixture of benzaldehyde (1.1 g, 1.14 mL, 0.01 mol), methyl acrylate (2.05 g, 2.15 mL, 0.023 mol) and DABCO (0.87 g, 0.0077 mol) in chloroform (5 mL) was stirred at room temperature for 7 d. The reaction mixture was quenched with 10% aqueous hydrochloric acid (50 mL) and washed repeatedly with water. The chloroform extract was then dried, concentrated, and purified by column chromatography (hexane/EtOAc 8:2, v/v) to afford **1a** as colourless oil (1.64 gm, 85%).

### Typical procedure for **3a–q**

A solution of AzPS (2 equiv) in acetonitrile (5 mL) was added to a solution of the Morita–Baylis–Hillman adduct **1a** (1 equiv) in acetonitrile (3 mL). The reaction mixture was then stirred for an hour, and 1.2 equiv of propargyl alcohol (**2a**) and CuI (5 mol %) were added. The reaction mixture was stirred for another 4 hours, followed by TLC analysis. After the completion of the reaction, the solution was concentrated, diluted, and extracted with EtOAc. The combined extracts were washed with brine, filtered through a celite bed, and dried over anhydrous Na_2_SO_4_. Thereafter, the solvent was removed, and the isolated crude oily product was purified over silica gel (CHCl_3_/MeOH) to obtain **3a** as a white solid.

### Typical procedure for **4a–c**

To a mixture of the Morita–Baylis–Hillman adduct (1 equiv) and AzPS (2 equiv) in acetonitrile (3 mL), HBr (2 equiv) was added carefully at room temperature. After 2 hours, the reaction mixture was quenched with water (20 mL) and then extracted with ethyl acetate. The organic layer was washed with brine and dried over anhydrous MgSO_4_. The removal of the solvent in vacuo afforded the crude product, which was purified over silica gel (using hexane/EtOAc) to acquire **4a** as colorless crystals.

## Supporting Information

File 1 Compound characterization data and NMR spectra.
