# Supplementary material for: Azidophosphonium salt-directed chemoselective synthesis of (E)/(Z)-cinnamyl-1H-triazoles and regiospecific access to bromomethylcoumarins from Morita–Baylis–Hillman adducts
Source: Beilstein J Org Chem. 2020 Jul 1;16:1579–87. doi: 10.3762/bjoc.16.130 (PMC7356223; doi:10.3762/bjoc.16.130)
Supplement: File 1 — Compound characterization data and NMR spectra. [file Beilstein_J_Org_Chem-16-1579-s001.pdf]

BEILSTEIN JOURNAL OF ORGANIC CHEMISTRY

Supporting Information

for

Azidophosphonium salt-directed chemoselective synthesis of (*E*)/(*Z*)-cinnamyl-1*H*-triazoles and regiospecific access to bromomethylcoumarins from Morita–Baylis–Hillman adducts

Soundararajan Karthikeyan, Radha Krishnan Shobana, Kamarajapurathu Raju Subimol, J. Helen Ratna Monica and Ayyanoth Karthik Krishna Kumar

Beilstein J. Org. Chem. **2020,** *16,* 1579–1587. doi:10.3762/bjoc.16.130

Compound characterization data and NMR spectra

License and Terms: This is a supporting information file under the terms of the Creative Commons Attribution License (http://creativecommons.org/licenses/by/4.0). Please note that the reuse, redistribution and reproduction in particular requires that the authors and source are credited. The license is subject to the *Beilstein Journal of Organic Chemistry* terms and conditions: (https://www.beilstein-journals.org/bjoc)

Table of Contents

1 Structure of the Morita–Baylis–Hillman–Adducts **1a**–**o** S1

2 Copies of ^{1}H NMR and ^{13}C NMR spectra for compounds **3a**–**q** S2

3 Copies of ^{1}H NMR and ^{13}C NMR spectra for compounds **4a**–**c** S36

4 Spectral data of (*E*)/(*Z*)-cinnamyl-1*H*-triazoles **3a**–**q** S42

5 Spectral data of 3-(bromomethyl)-2*H*-chromen-2-one **4a**–**c** S47

Structure of the Morita–Baylis–Hillman Adducts 1a–o

Morita–Baylis–Hillman adducts 1a–l utilised for the analysis

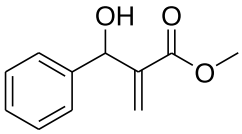
1a

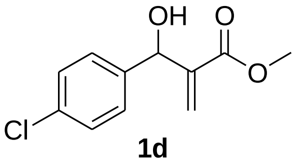
1d

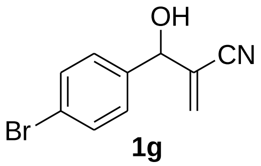
1g

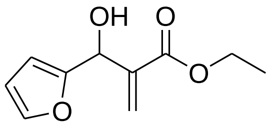
1j

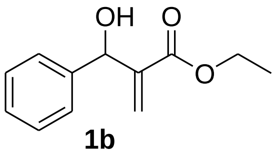
1b

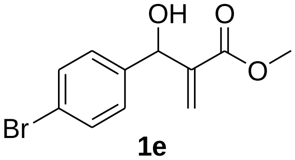
1e

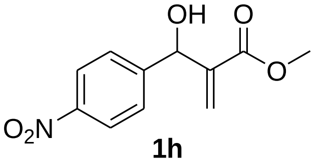
1h

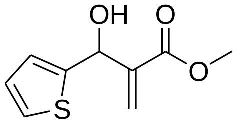
1k

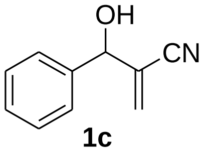
1c

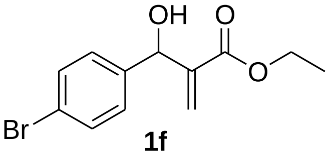
1f

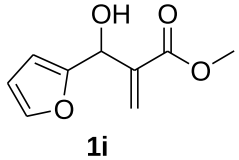
1i

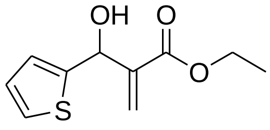
1l

Morita–Baylis–Hillman adducts 1m–o utilised for the analysis

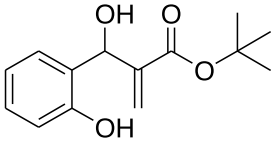

1m

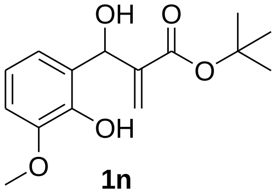

1n

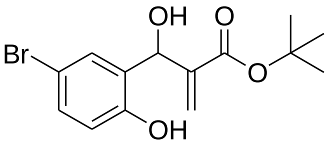

1o

Copies of ^{1}H NMR and ^{13}C NMR spectra for compounds 3a–q

Proton NMR spectrum of (*E*)-methyl 2-((4-hydroxymethyl)-1*H*-1,2,3-triazol-1-yl)methyl)-3-phenylacrylate (3a)

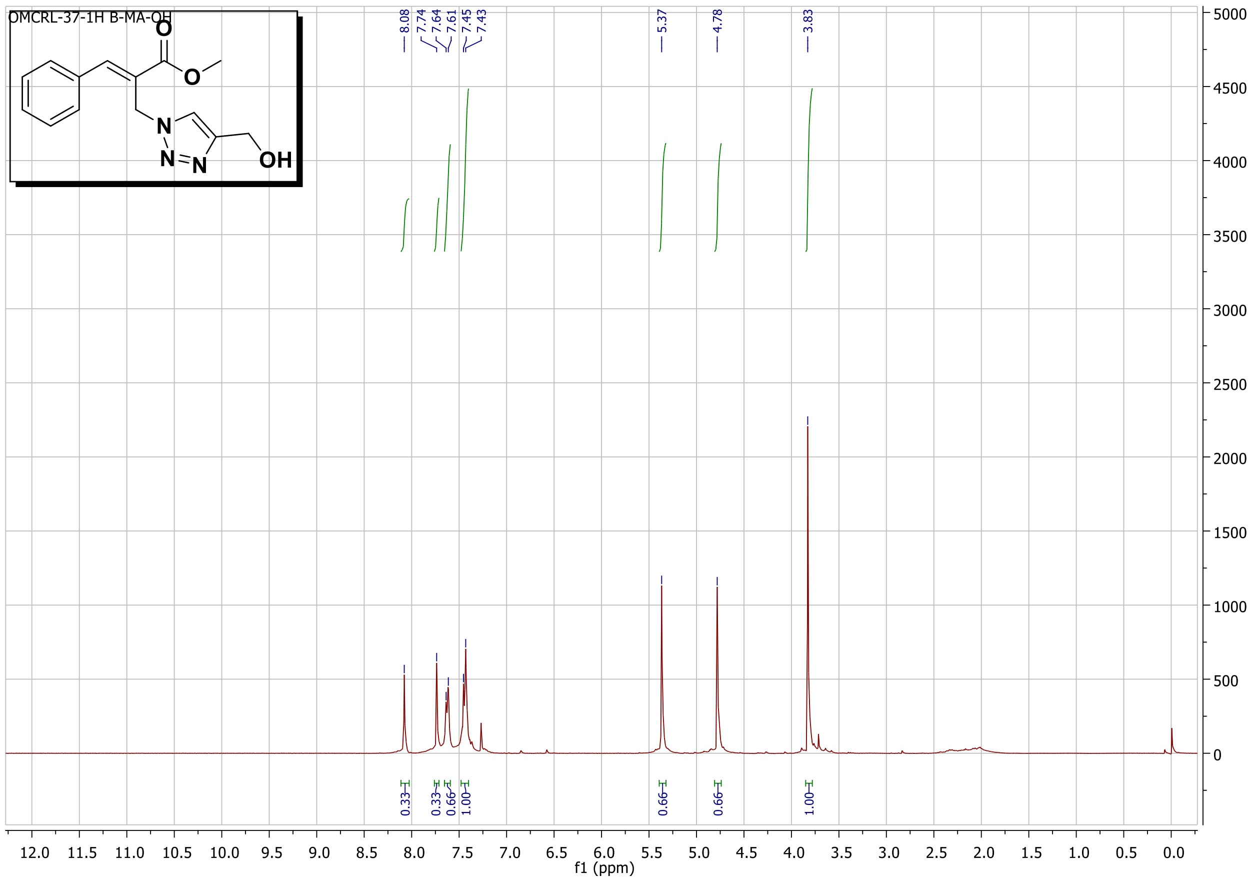

Figure S1 ^{1}H NMR spectrum of (*E*)-methyl 2-((4-hydroxymethyl)-1*H*-1,2,3-triazol-1-yl)methyl)-3-phenylacrylate (**3a**)

Carbon NMR spectrum of (*E*)-methyl 2-((4-hydroxymethyl)-1*H*-1,2,3-triazol-1-yl)methyl)-3-phenylacrylate (3a)

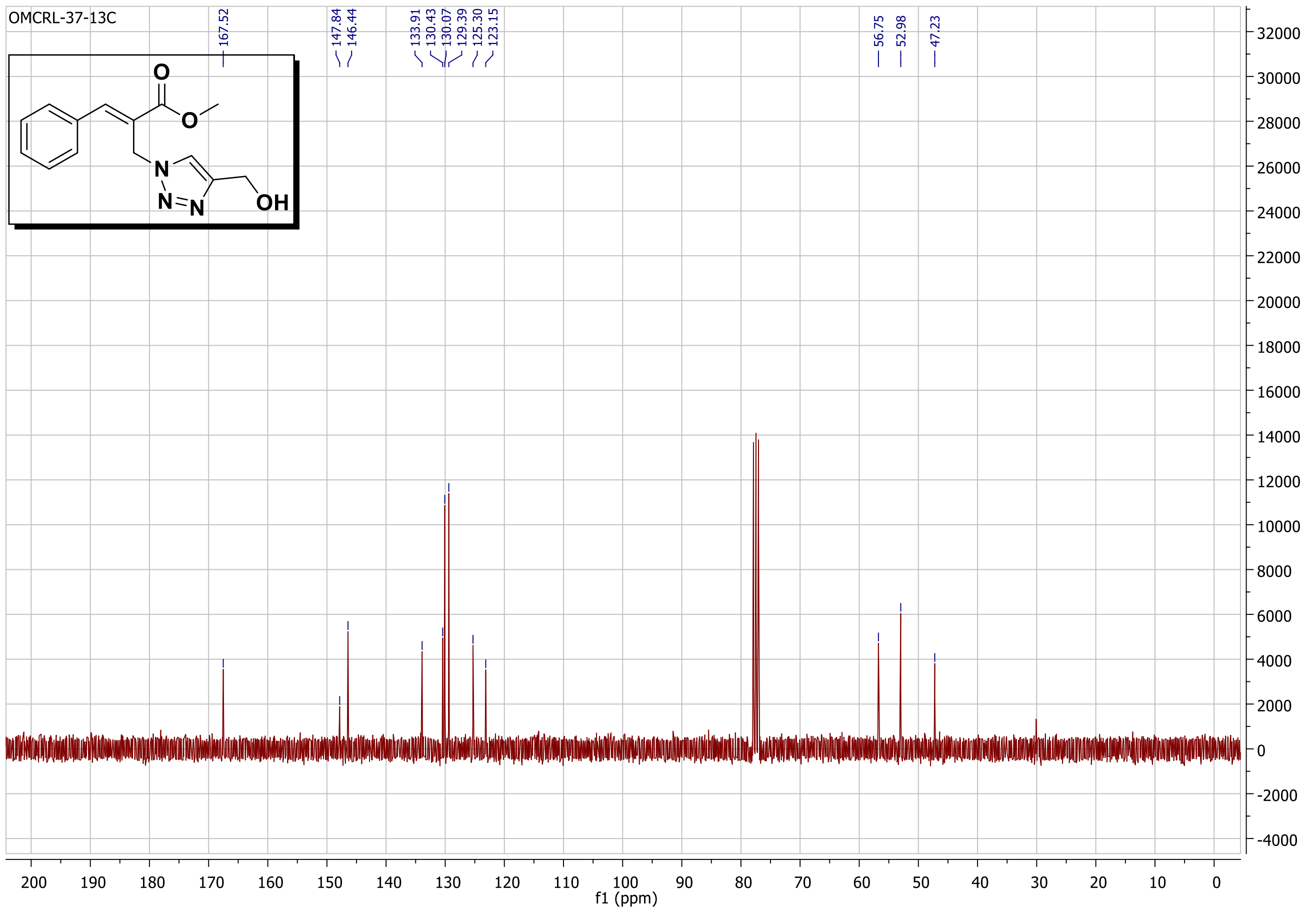

Figure S2 ^{13}C NMR spectrum of (*E*)-methyl 2-((4-hydroxymethyl)-1*H*-1,2,3-triazol-1-yl)methyl)-3-phenylacrylate (**3a**)

Proton NMR spectrum of (*E*)-methyl 3-phenyl-2-((4-phenyl-1*H*-1,2,3-triazol-1-yl)methyl)acrylate (3b)

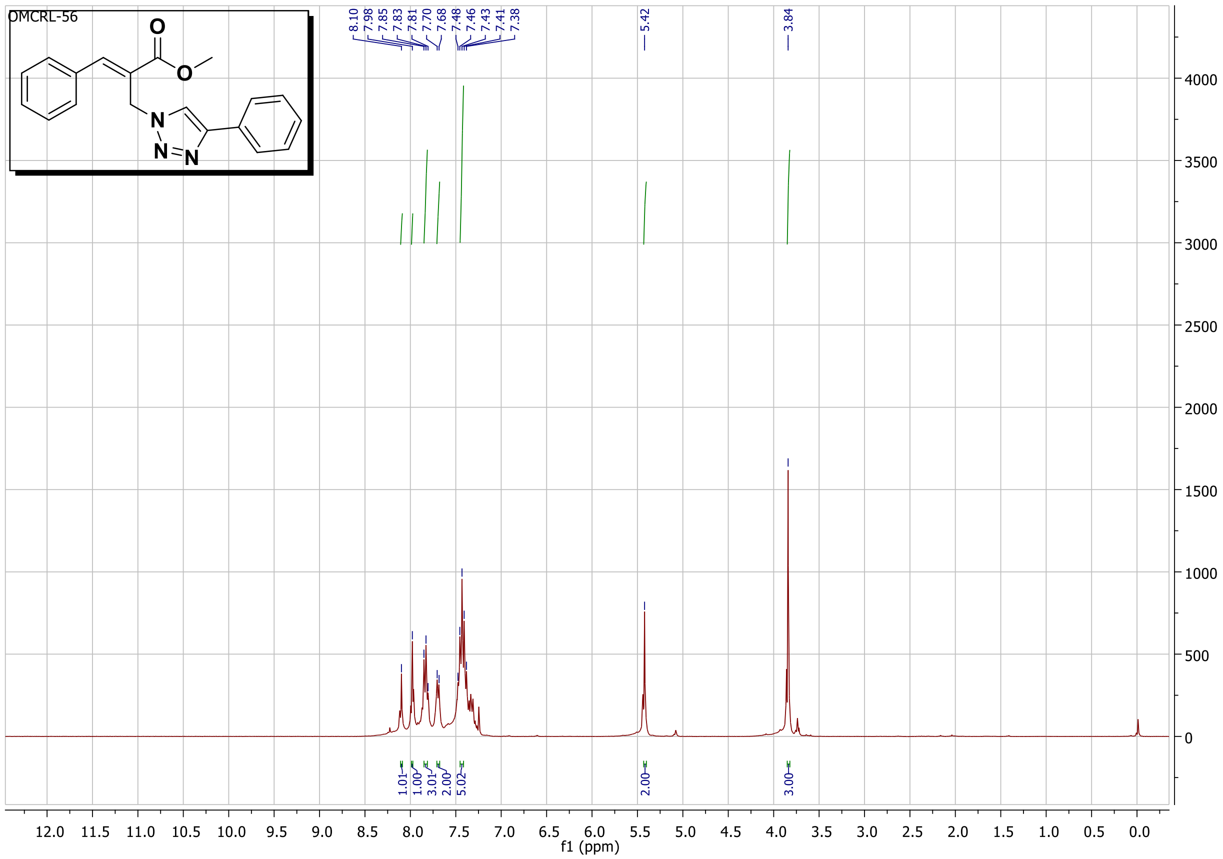

Figure S3 ^{1}H NMR spectrum of (*E*)-methyl 3-phenyl-2-((4-phenyl-1*H*-1,2,3-triazol-1-yl)methyl)acrylate (**3b**)

Carbon NMR spectrum of (*E*)-methyl 3-phenyl-2-((4-phenyl-1*H*-1,2,3-triazol-1-yl)methyl)acrylate (3b)

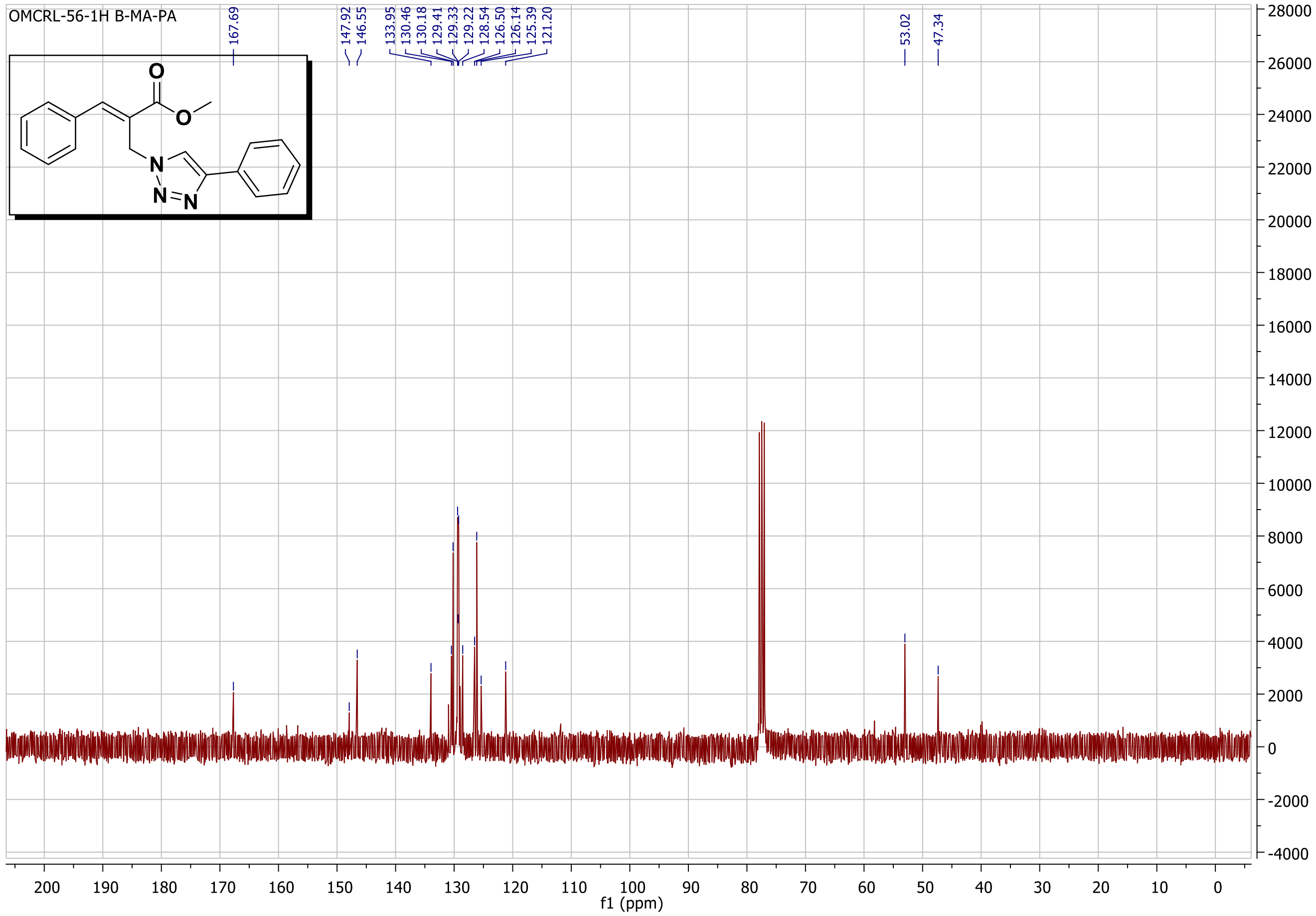

Figure S4 ^{13}C NMR spectrum of (*E*)-methyl 3-phenyl-2-((4-phenyl-1*H*-1,2,3-triazol-1-yl)methyl)acrylate (**3b**)

Proton NMR spectrum of (*E*)-ethyl 2-((4-hydroxymethyl)-1*H*-1,2,3-triazol-1-yl)methyl)-3-phenylacrylate (3c)

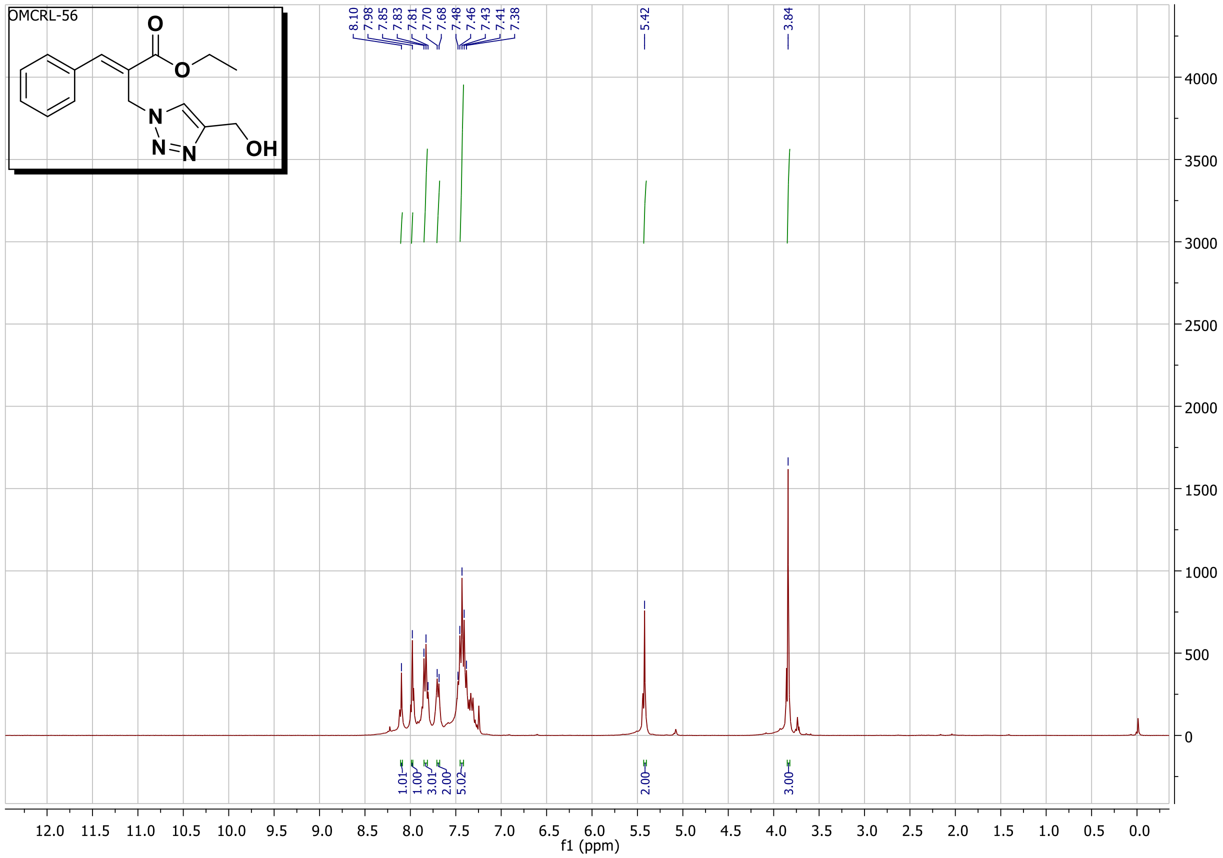

Figure S5 ^{1}H NMR spectrum of (*E*)-ethyl 2-((4-hydroxymethyl)-1*H*-1,2,3-triazol-1-yl)methyl)-3-phenylacrylate (**3c**)

Carbon NMR spectrum of (*E*)-ethyl 2-((4-hydroxymethyl)-1*H*-1,2,3-triazol-1-yl)methyl)-3-phenylacrylate (3c)

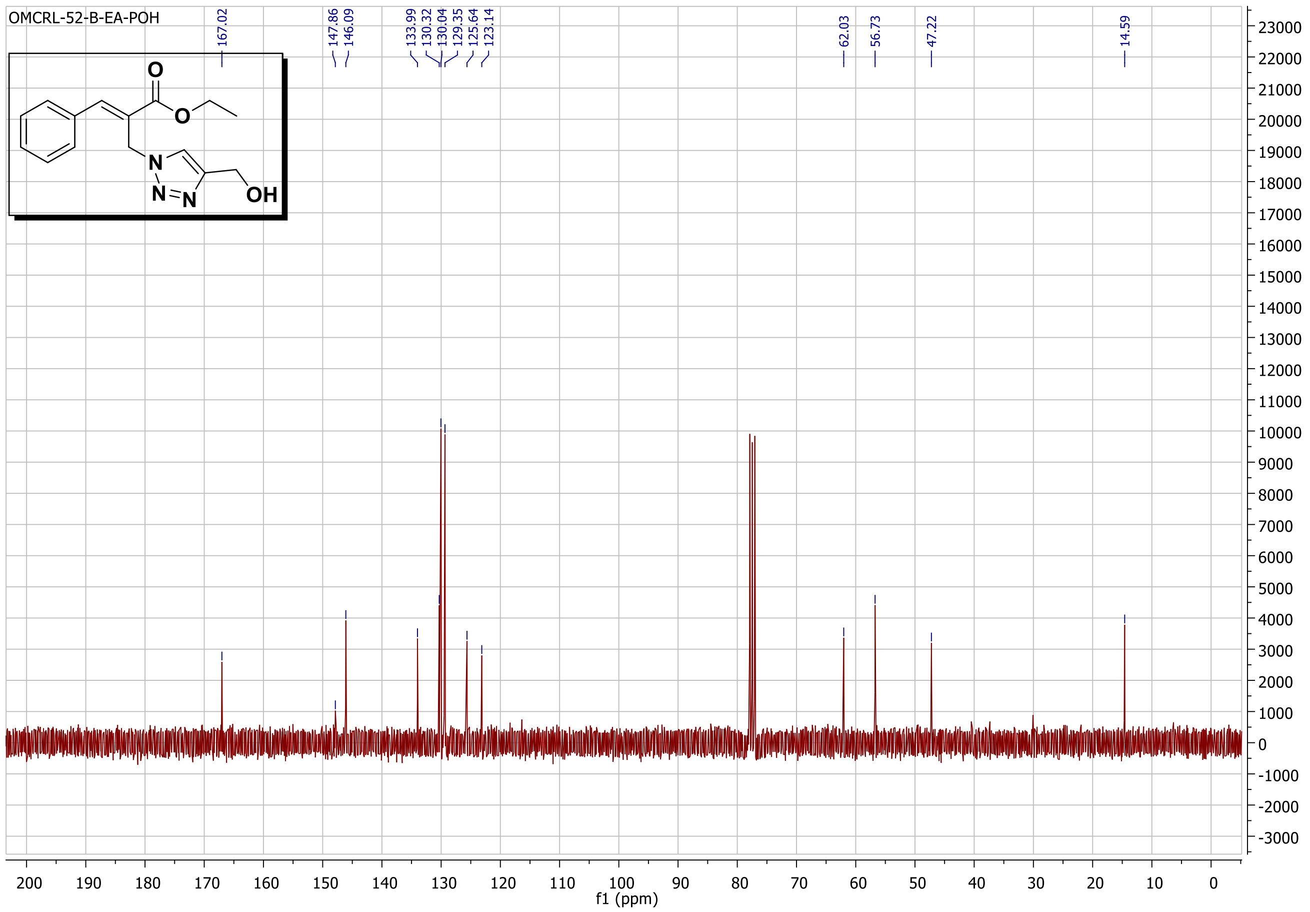

Figure S6 ^{13}C NMR spectrum of (*E*)-ethyl 2-((4-hydroxymethyl)-1*H*-1,2,3-triazol-1-yl)methyl)-3-phenylacrylate (**3c**)

Proton NMR spectrum of (*E*)-ethyl 3-phenyl-2-((4-phenyl-1*H*-1,2,3-triazol-1-yl)methyl)acrylate (3d)

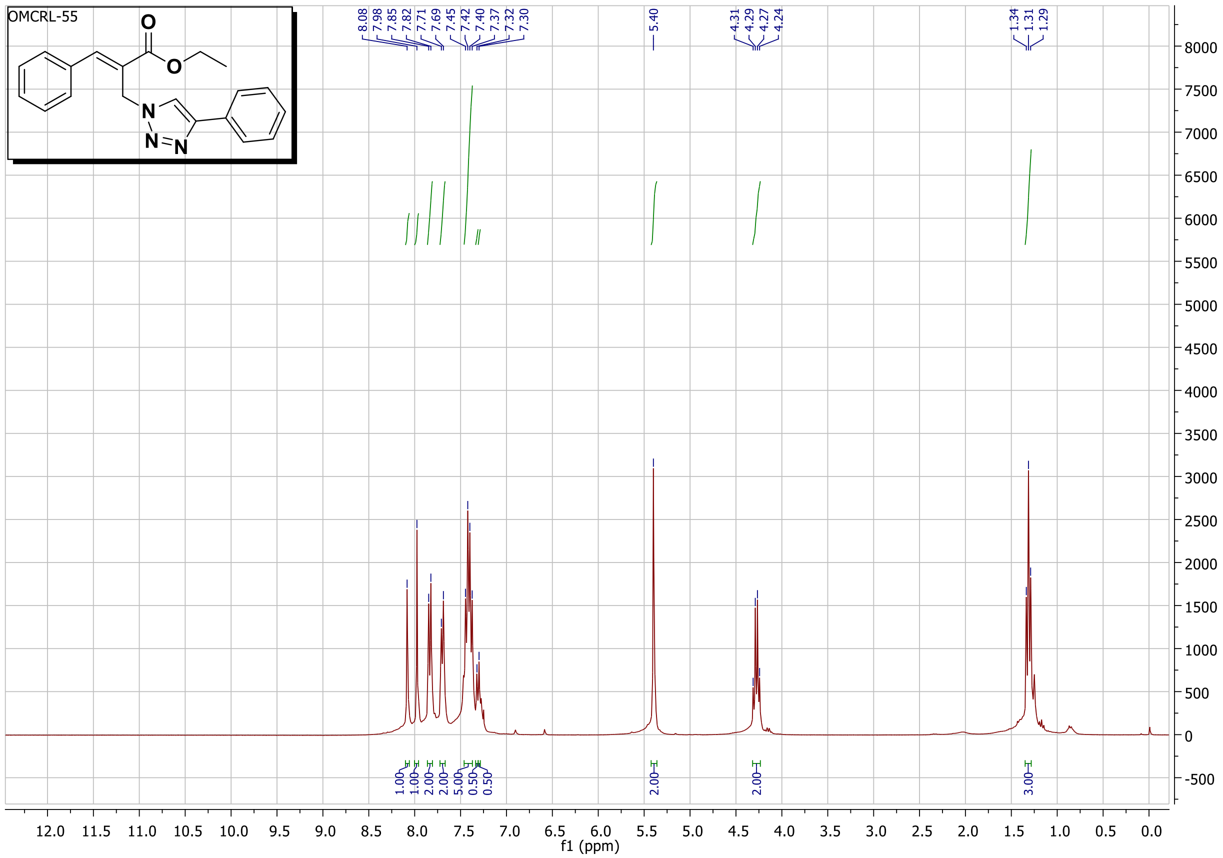

Figure S7 ^{1}H NMR spectrum of (*E*)-ethyl 3-phenyl-2-((4-phenyl-1*H*-1,2,3-triazol-1-yl)methyl)acrylate (**3d**)

Carbon NMR spectrum of (*E*)-ethyl 3-phenyl-2-((4-phenyl-1*H*-1,2,3-triazol-1-yl)methyl)acrylate (3d)

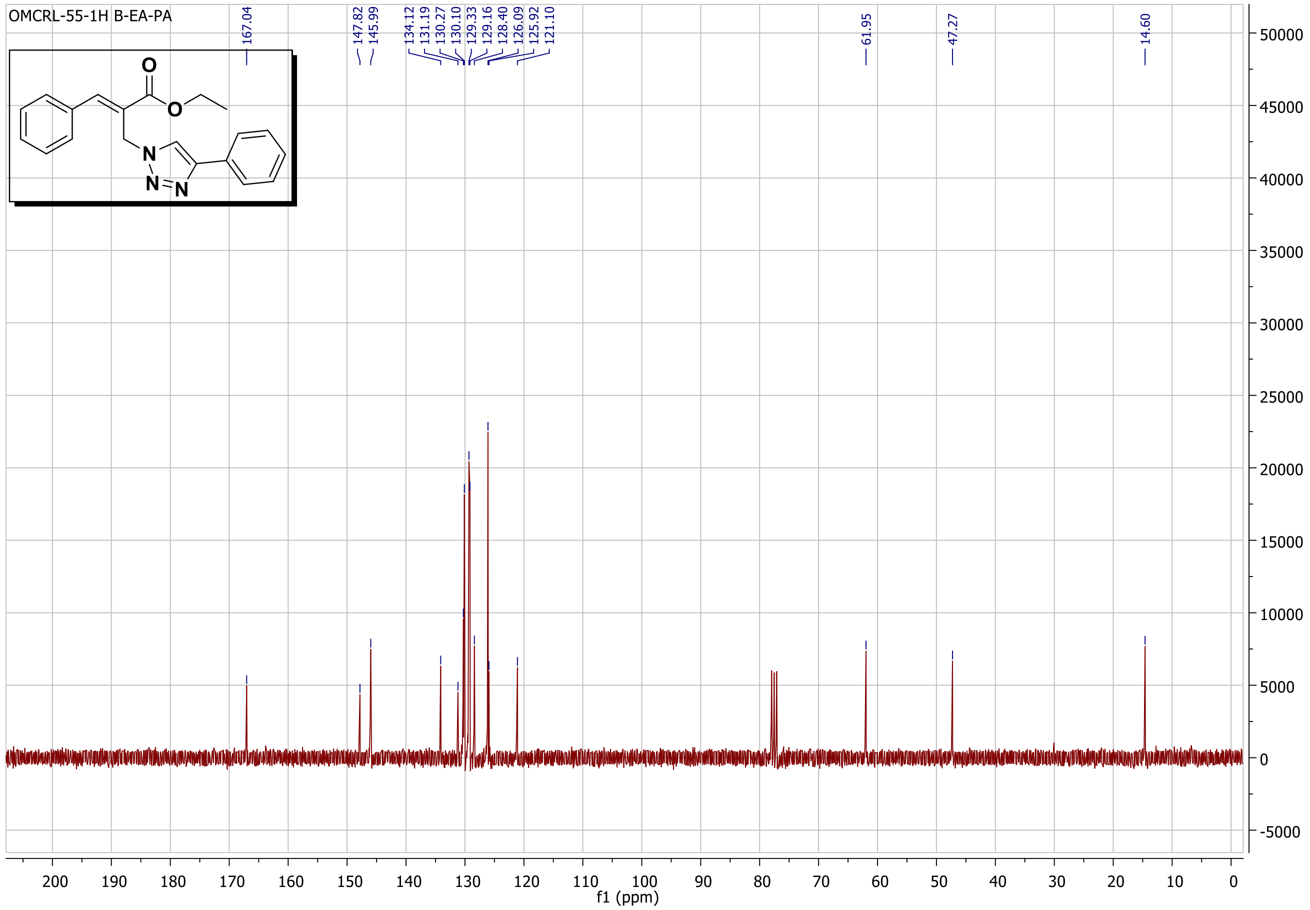

Figure S8 ^{13}C NMR spectrum of (*E*)-ethyl 3-phenyl-2-((4-phenyl-1*H*-1,2,3-triazol-1-yl)methyl)acrylate (**3d**)

Proton NMR spectrum of (*Z*)-2-((4-(hydroxymethyl)-1*H*-1,2,3-triazol-1-yl)methyl)-3-phenylacrylonitrile (3e)

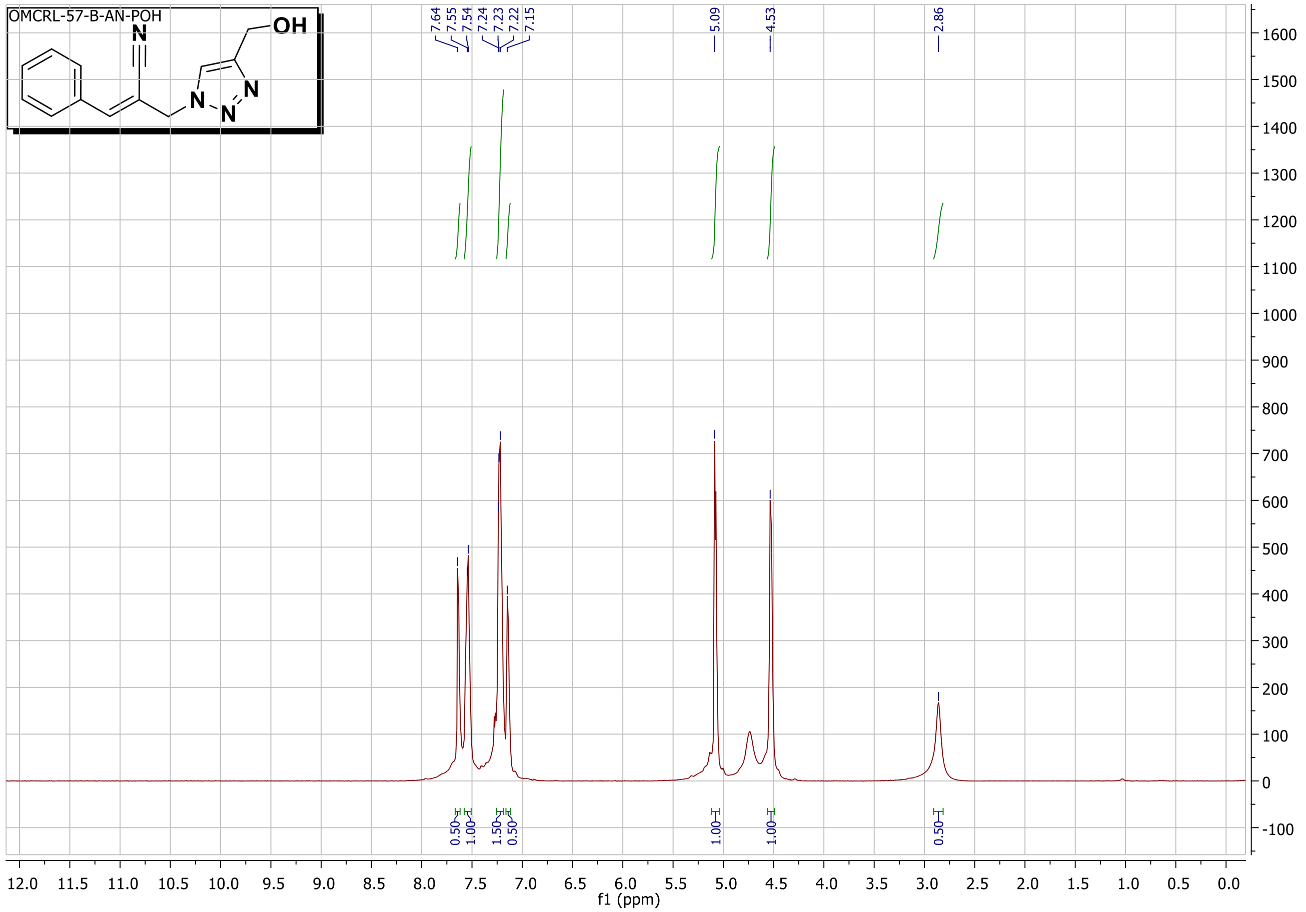

Figure S9 ^{1}H NMR spectrum of (*Z*)-2-((4-(hydroxymethyl)-1*H*-1,2,3-triazol-1-yl)methyl)-3-phenylacrylonitrile (**3e**)

Carbon NMR spectrum of (*Z*)-2-((4-(hydroxymethyl)-1H-1,2,3-triazol-1-yl)methyl)-3-phenylacrylonitrile (3e)

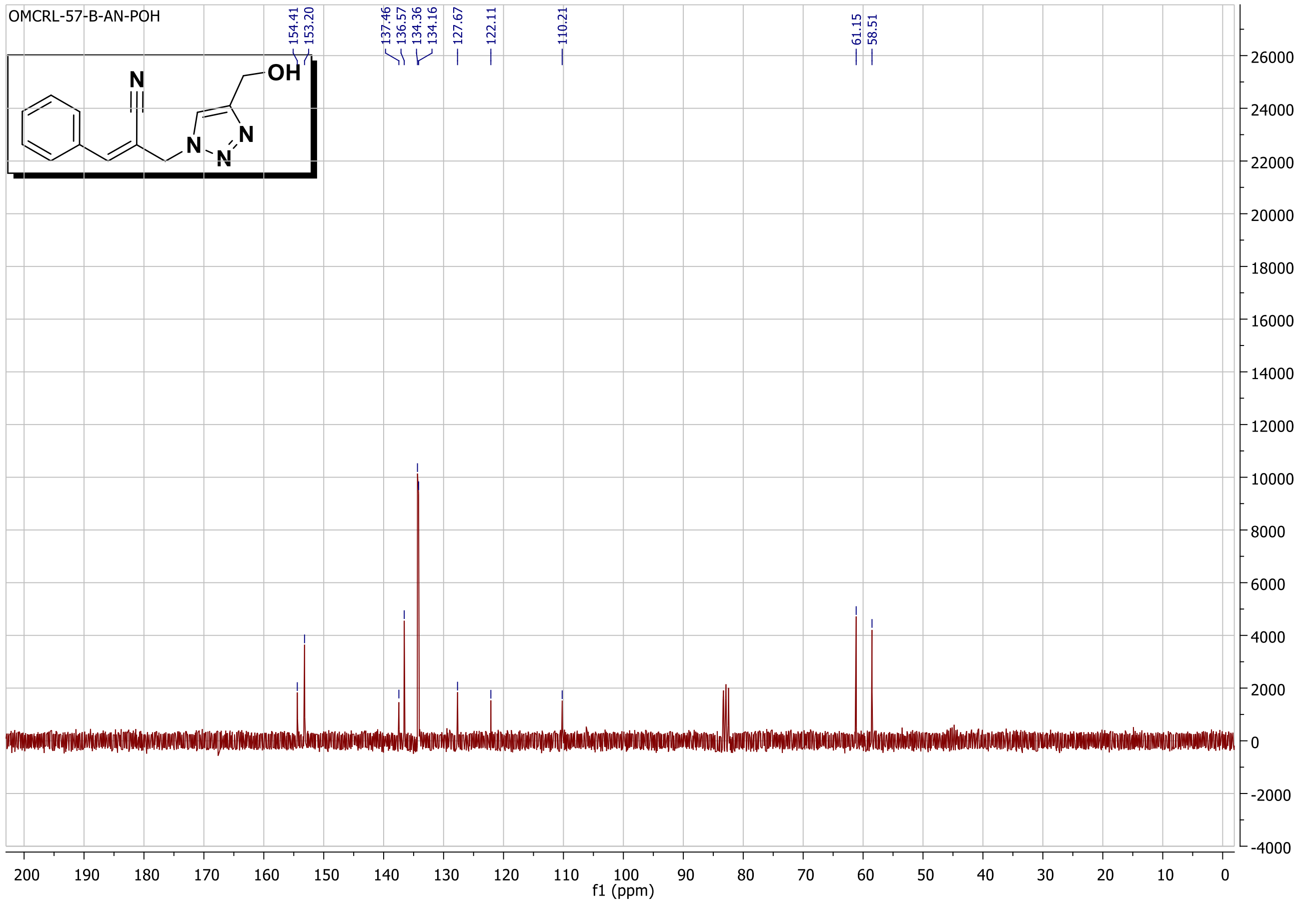

Figure S10 ^{13}C NMR spectrum of (*Z*)-2-((4-(hydroxymethyl)-1*H*-1,2,3-triazol-1-yl)methyl)-3-phenylacrylonitrile (**3e**)

Proton NMR spectrum of (*Z*)-3-phenyl-2-((4-phenyl-1*H*-1,2,3-triazol-1-yl)methyl)acrylonitrile (3f)

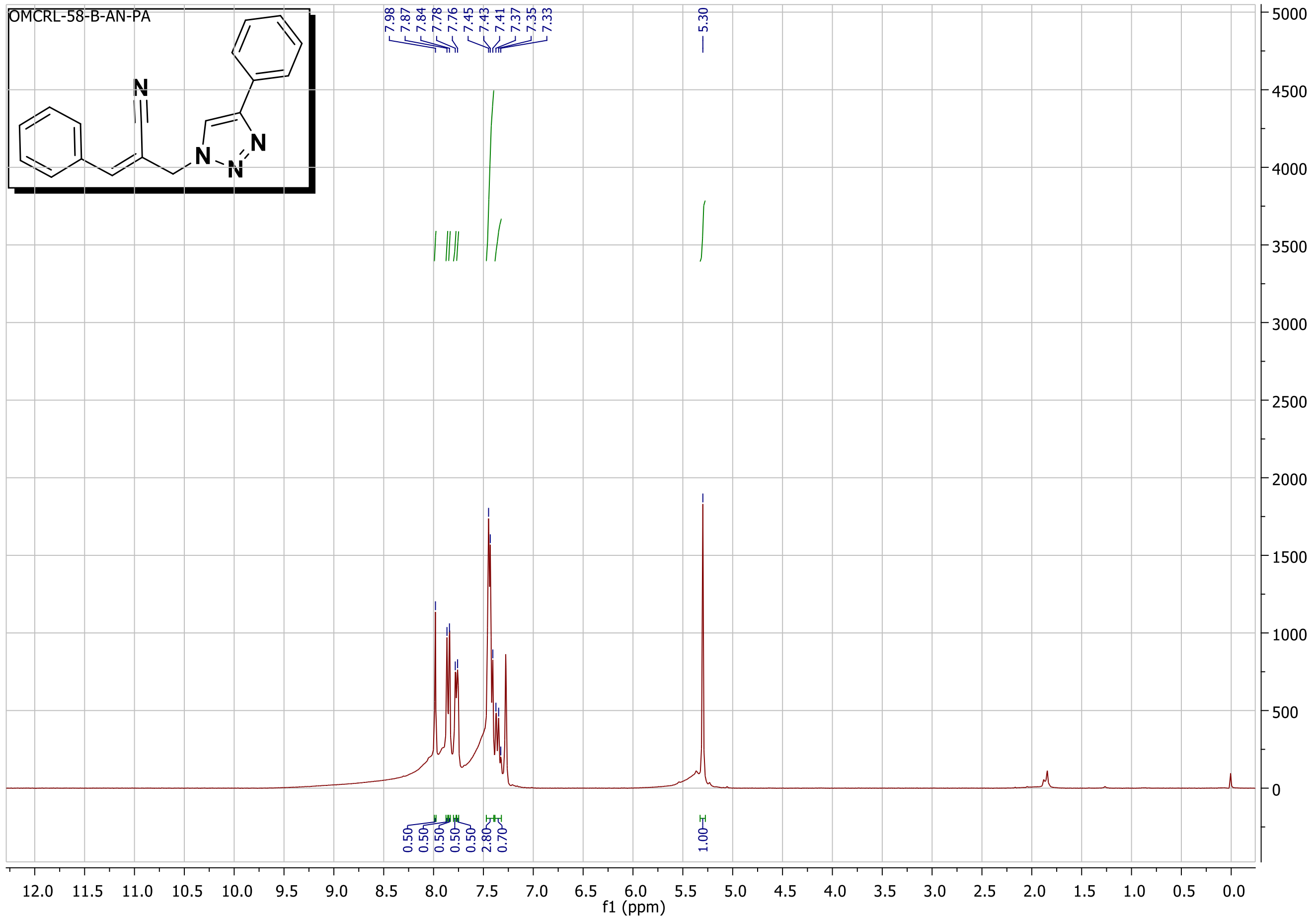

Figure S11 ^{1}H NMR spectrum of (*Z*)-3-phenyl-2-((4-phenyl-1*H*-1,2,3-triazol-1-yl)methyl)acrylonitrile (**3f**)

Carbon NMR spectrum of (*Z*)-3-phenyl-2-((4-phenyl-1*H*-1,2,3-triazol-1-yl)methyl)acrylonitrile (3f)

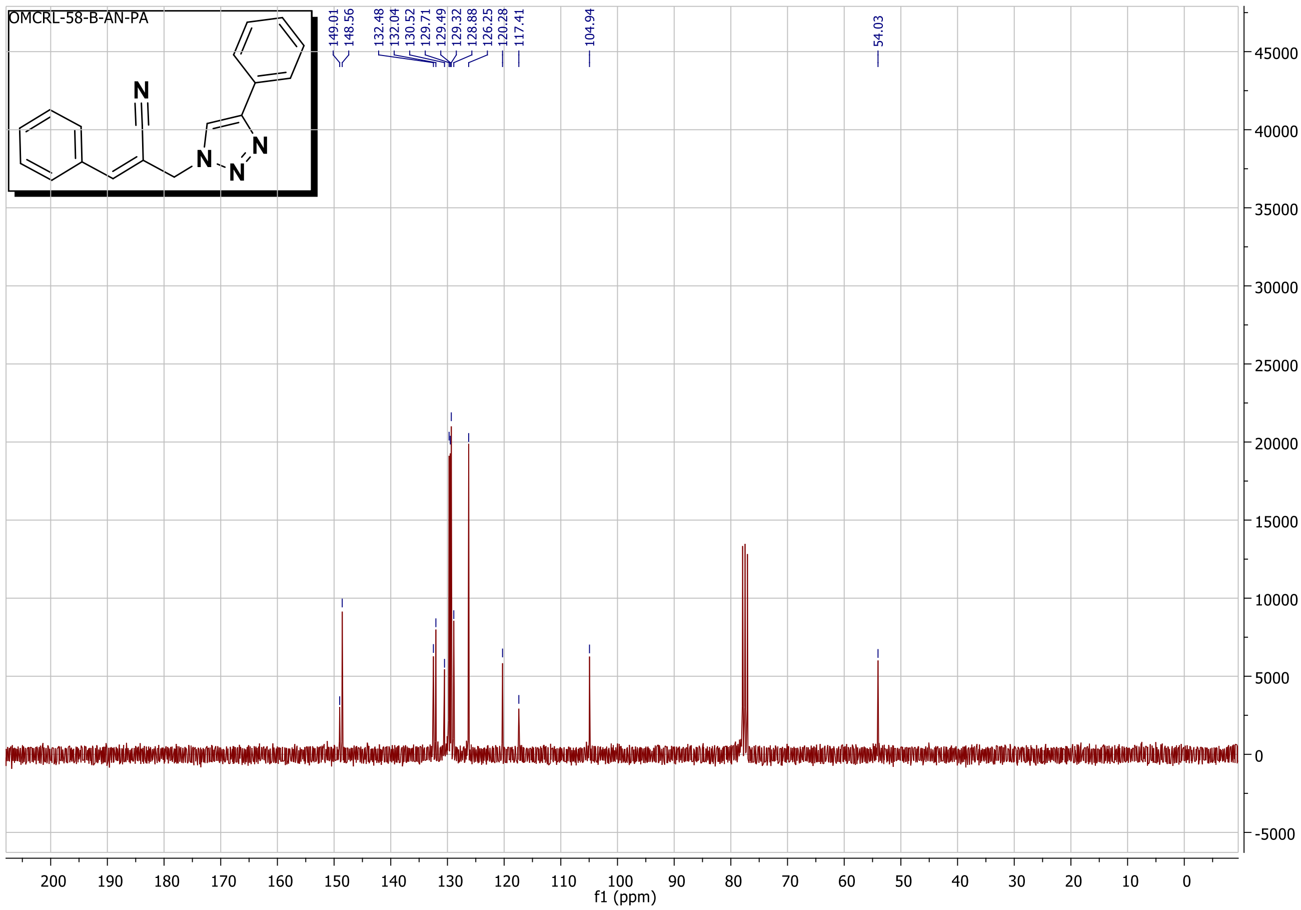

Figure S12 ^{13}C NMR spectrum of (*Z*)-3-phenyl-2-((4-phenyl-1*H*-1,2,3-triazol-1-yl)methyl)acrylonitrile (**3f**)

Proton NMR spectrum of (*E*)-methyl 3-(4-chlorophenyl)-2-((4-(hydroxymethyl)-1*H*-1,2,3-triazol-1-yl)methyl)acrylate (3g)

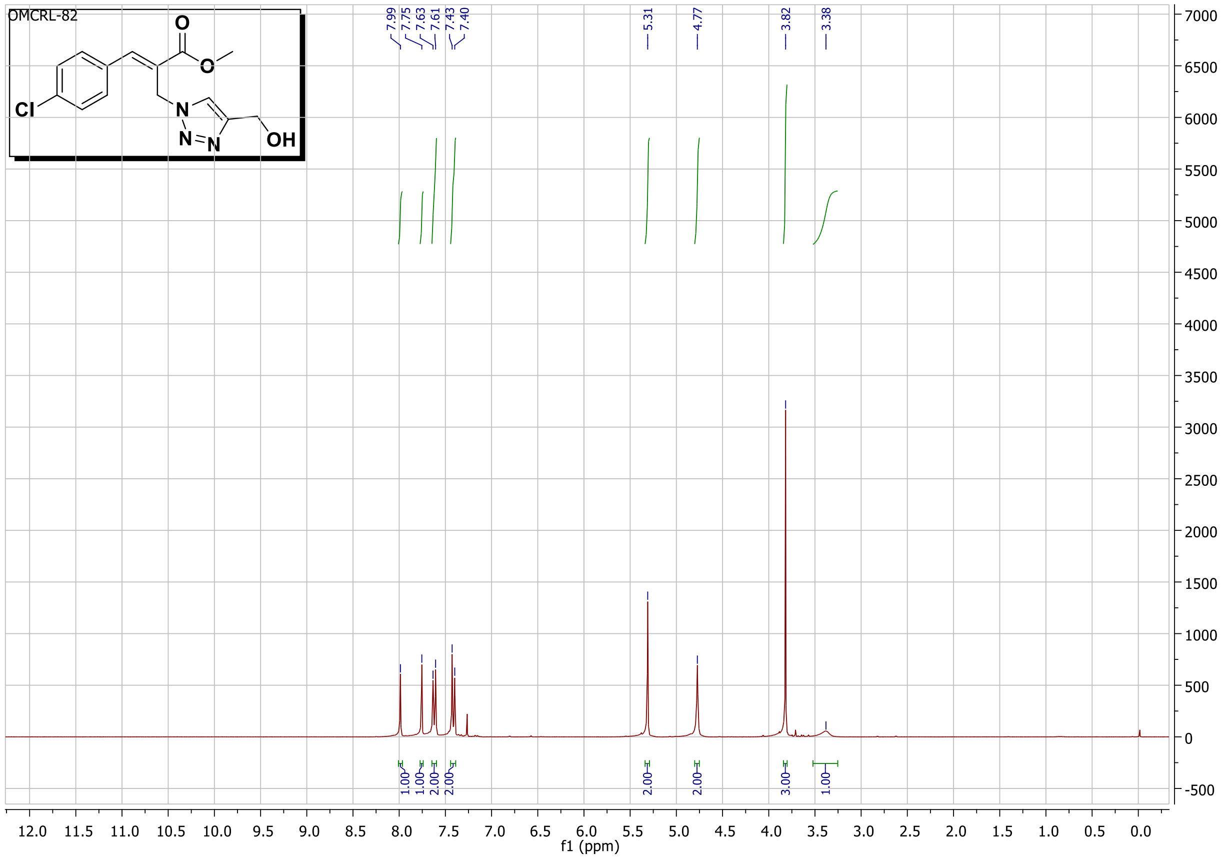

Figure S13 ^{1}H NMR spectrum of (*E*)-methyl 3-(4-chlorophenyl)-2-((4-(hydroxymethyl)-1*H*-1,2,3-triazol-1-yl)methyl)acrylate (**3g**)

Carbon NMR spectrum of (*E*)-methyl 3(4-chlorophenyl)-2-((4-(hydroxymethyl)-1*H*-1,2,3-triazol-1-yl)methyl)acrylate (3g)

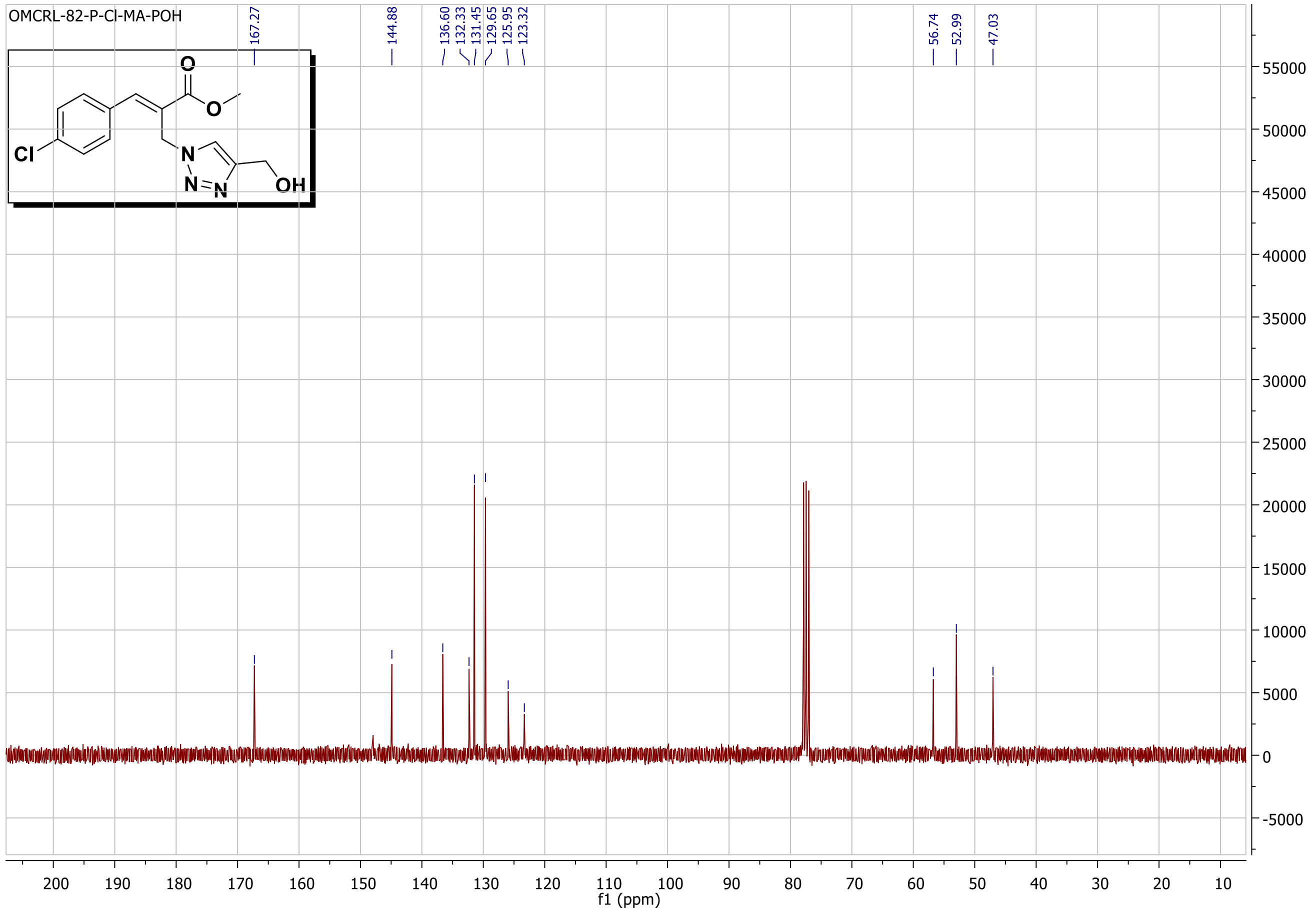

Figure S14 ^{13}C NMR spectrum of (*E*)-methyl 3(4-chlorophenyl)-2-((4-(hydroxymethyl)-1*H*-1,2,3-triazol-1-yl)methyl)acrylate (**3g**)

Proton NMR spectrum of (*E*)-methyl 3-(4-chlorophenyl)-2-((4-phenyl-1*H*-1,2,3-triazol-1-yl)methyl)acrylate (3h)

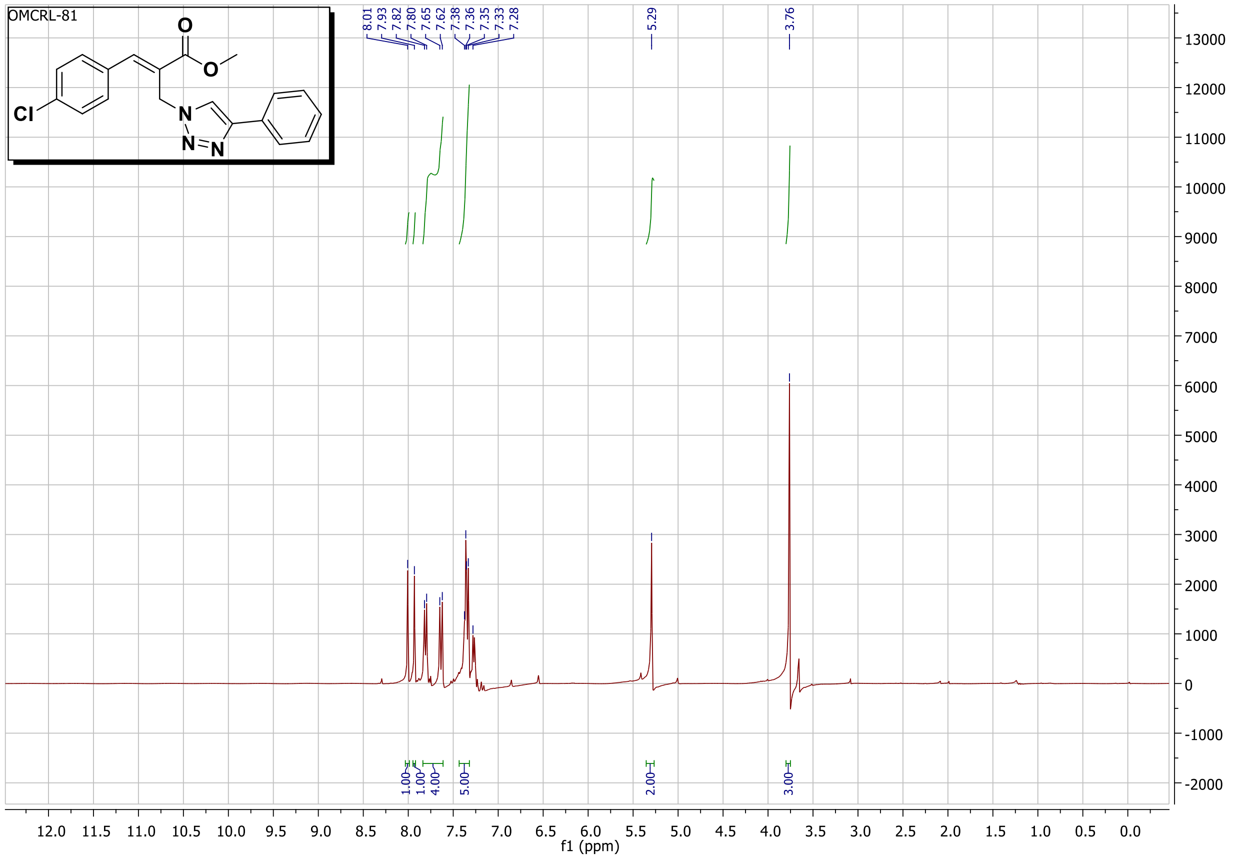

Figure S15 ^{1}H NMR spectrum of (*E*)-methyl 3-(4-chlorophenyl)-2-((4-phenyl-1*H*-1,2,3-triazol-1-yl)methyl)acrylate (**3h**)

Carbon NMR spectrum of (*E*)-methyl 3-(4-chlorophenyl)-2-((4-phenyl-1*H*-1,2,3-triazol-1-yl)methyl)acrylate (3h)

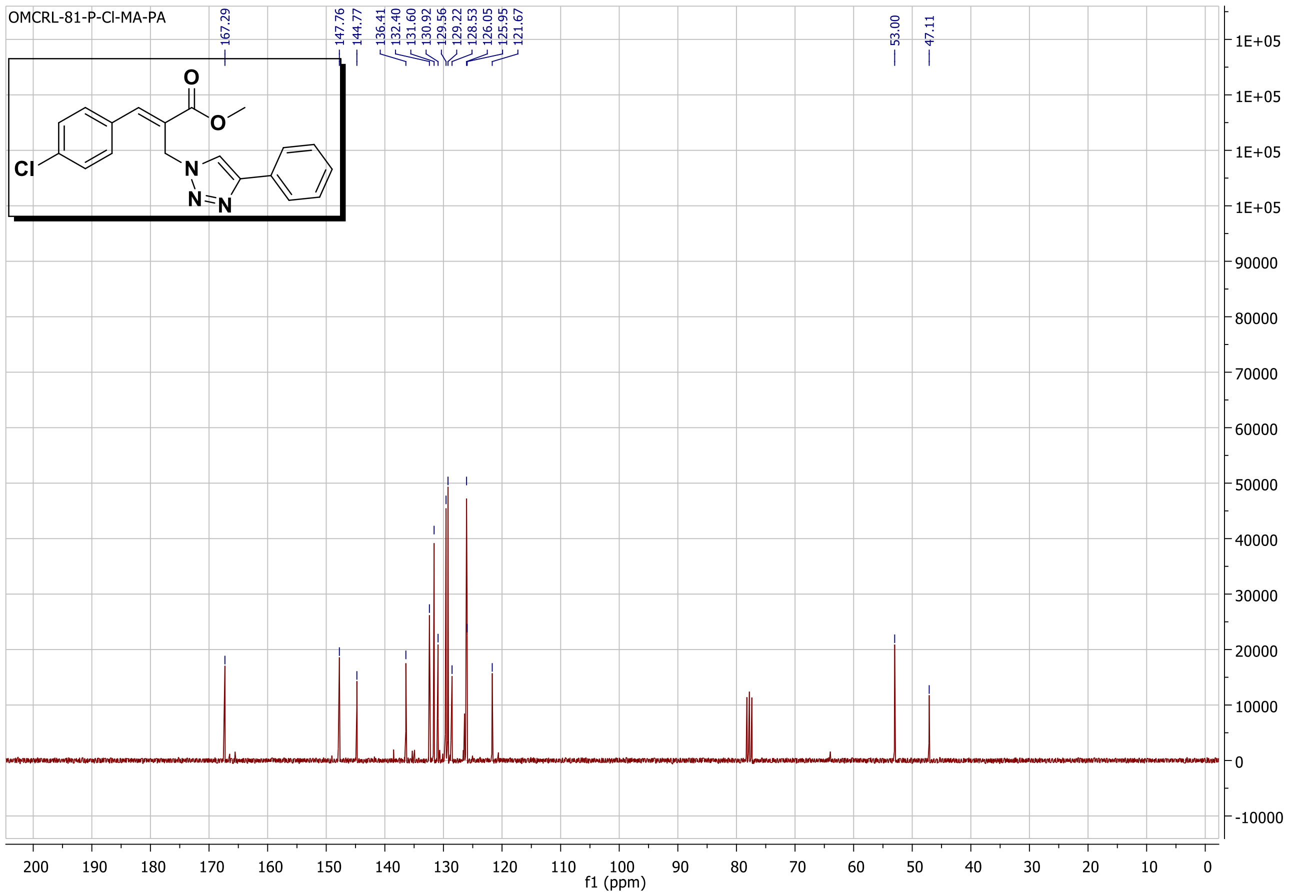

Figure S16 ^{13}C NMR spectrum of (*E*)-methyl 3-(4-chlorophenyl)-2-((4-phenyl-1*H*-1,2,3-triazol-1-yl)methyl)acrylate (**3h**)

Proton NMR spectrum of (*E*)-methyl 3-(4-bromophenyl)-2-((4-(hydroxymethyl)-1*H*-1,2,3-triazol-1-yl)methyl)acrylate (3i)

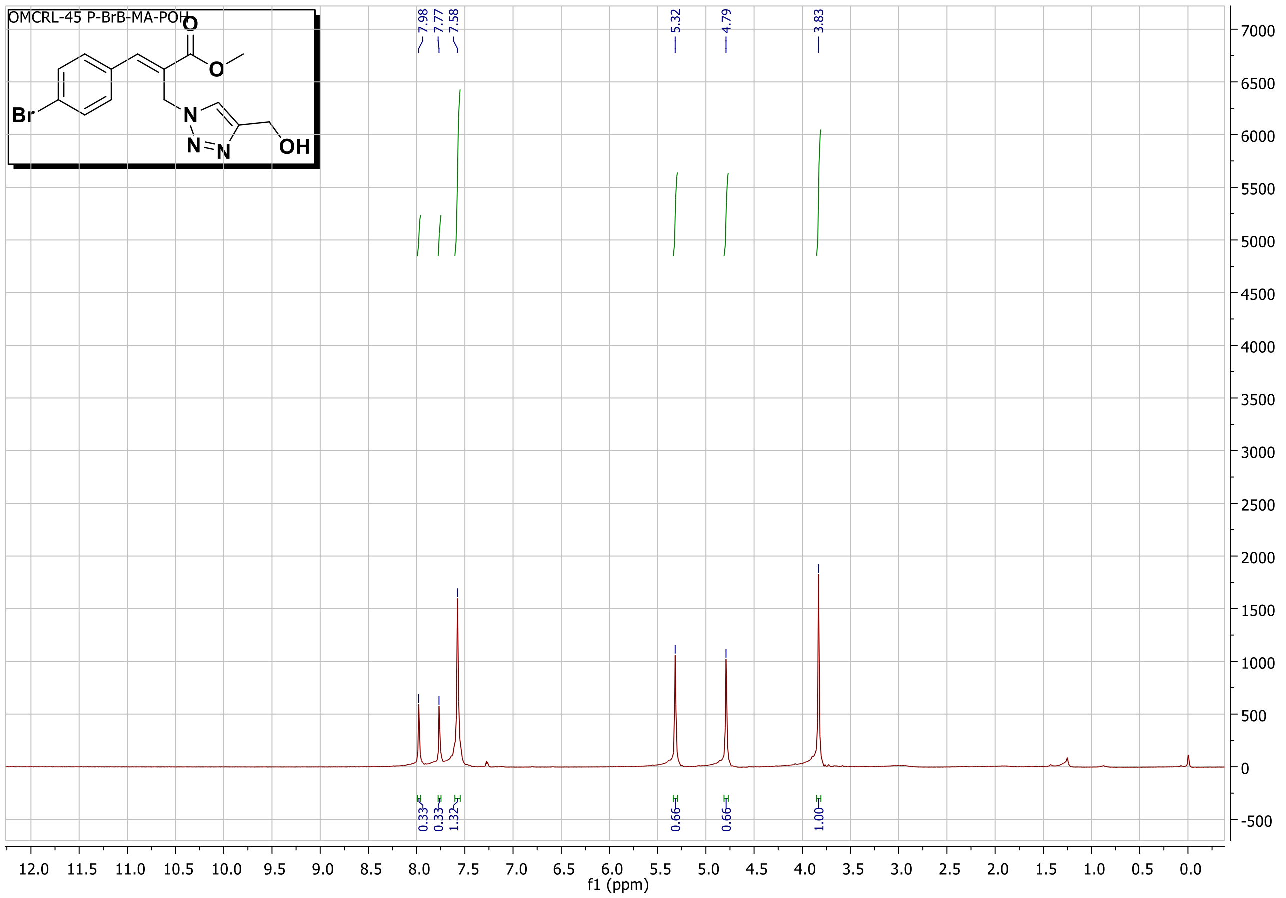

Figure S17 ^{1}H NMR spectrum of (*E*)-methyl 3-(4-bromophenyl)-2-((4-(hydroxymethyl)-1*H*-1,2,3-triazol-1-yl)methyl)acrylate (**3i**)

Carbon NMR spectrum of (*E*)-methyl 3-(4-bromophenyl)-2-((4-(hydroxymethyl)-1*H*-1,2,3-triazol-1-yl)methyl)acrylate (3i)

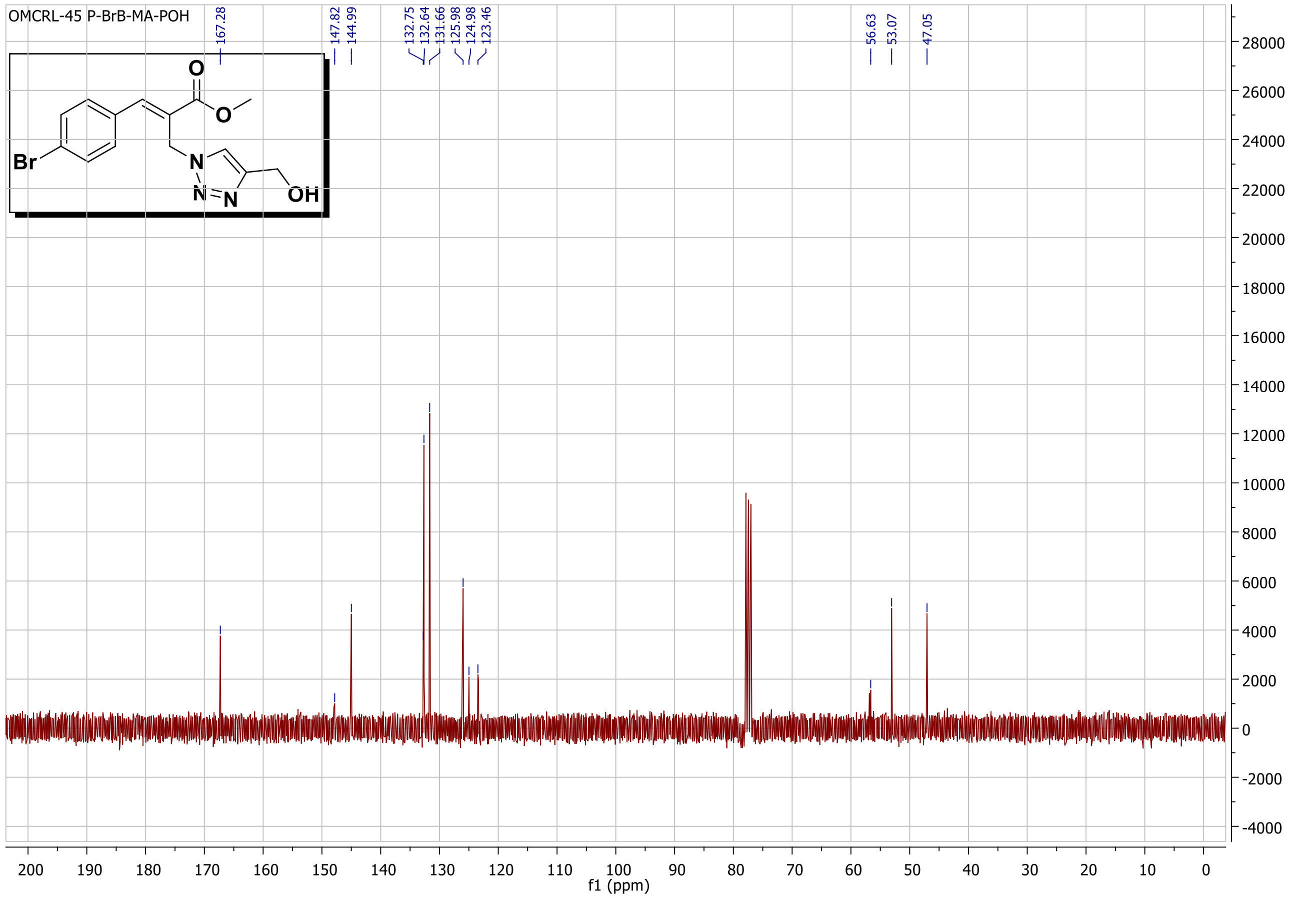

Figure S18 ^{13}C NMR spectrum of (*E*)-methyl 3-(4-bromophenyl)-2-((4-(hydroxymethyl)-1*H*-1,2,3-triazol-1-yl)methyl)acrylate (**3i**)

Proton NMR spectrum of (*E*)-methyl 3-(4-bromophenyl)-2-((4-phenyl-1*H*-1,2,3-triazol-1-yl)methyl)acrylate (3j)

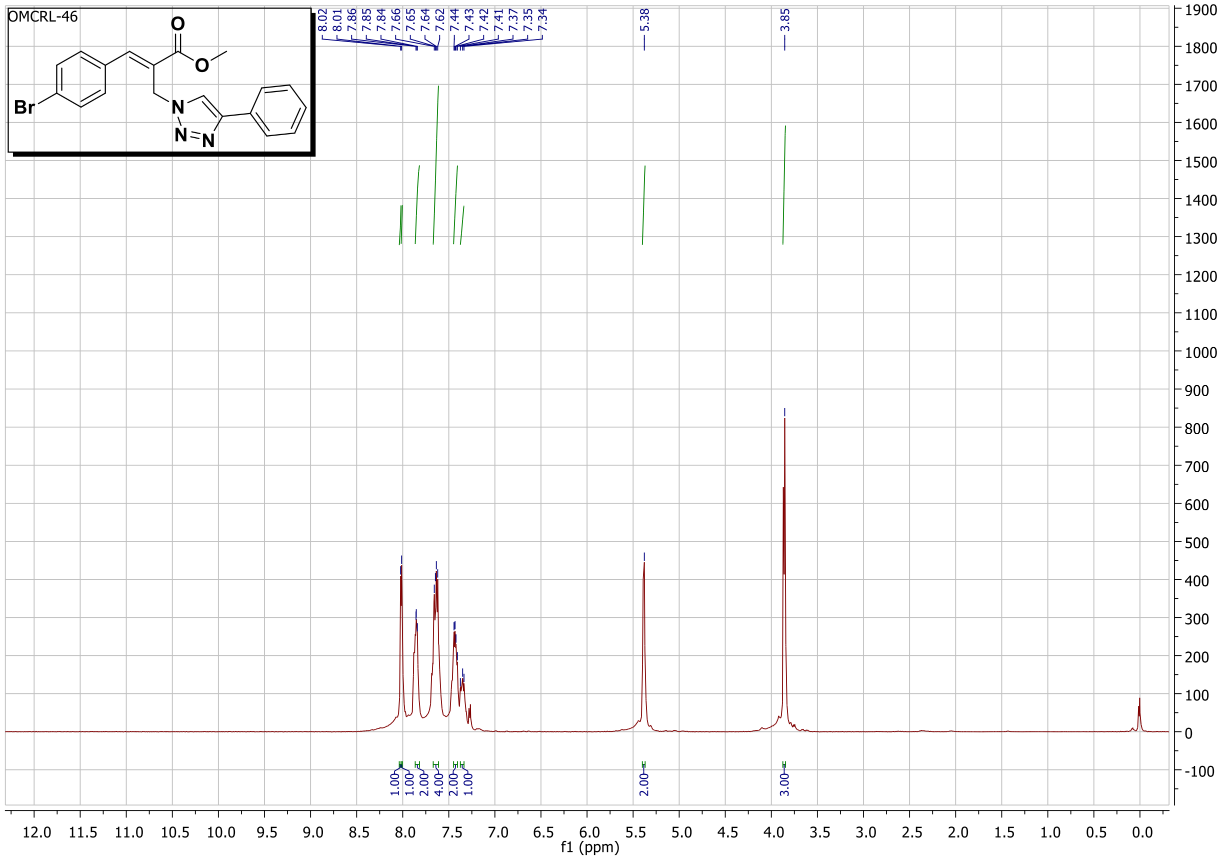

Figure S19 ^{1}H NMR spectrum of (*E*)-methyl 3-(4-bromophenyl)-2-((4-phenyl-1*H*-1,2,3-triazol-1-yl)methyl)acrylate (**3j)**

Carbon NMR spectrum of (*E*)-methyl 3-(4-bromophenyl)-2-((4-phenyl-1*H*-1,2,3-triazol-1-yl)methyl)acrylate (3j)

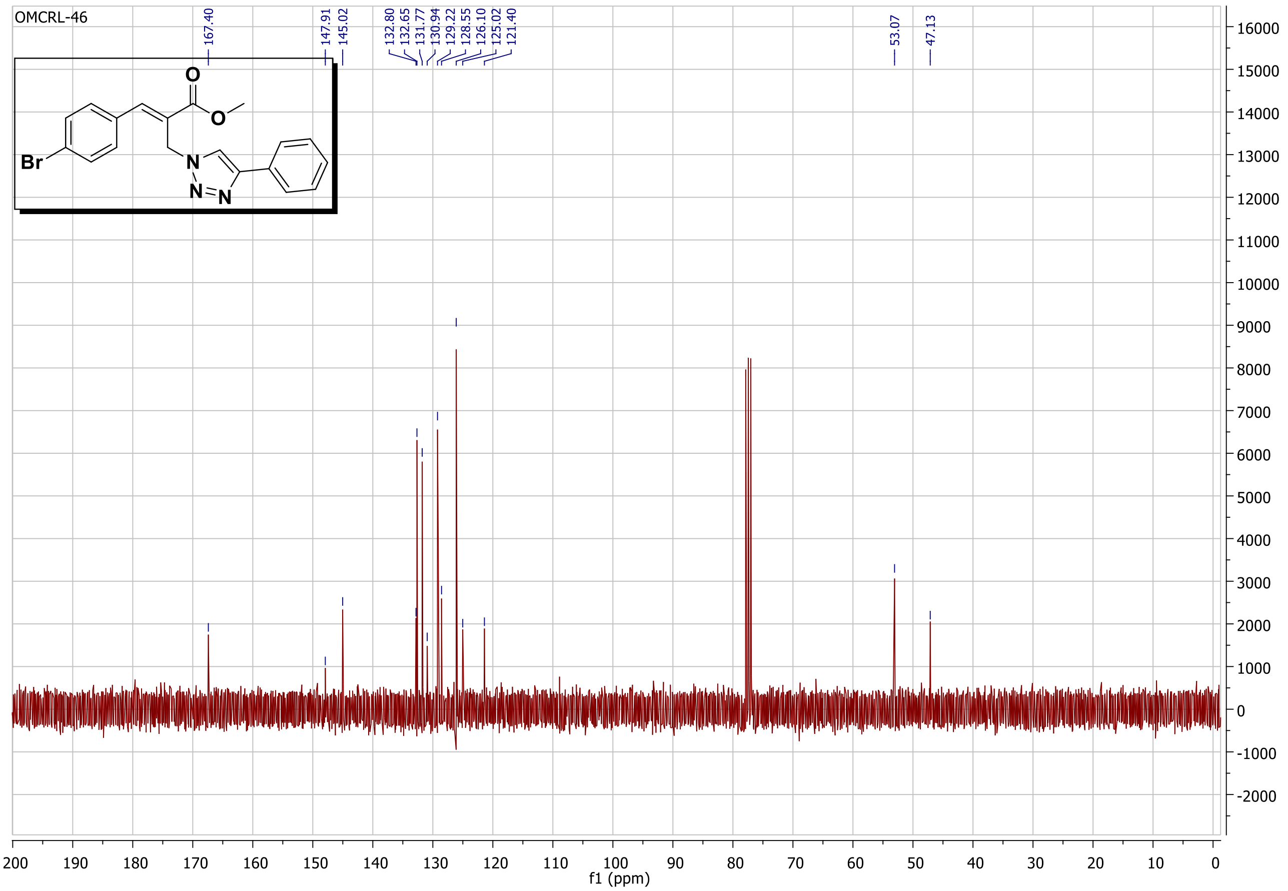

Figure S20 ^{13}C NMR spectrum of (*E*)-methyl 3-(4-bromophenyl)-2-((4-phenyl-1*H*-1,2,3-triazol-1-yl)methyl)acrylate (**3j**)

Proton NMR spectrum of (*E*)-ethyl 3-(4-bromophenyl)-2-((4-(hydroxymethyl)-1*H*-1,2,3-triazol-1-yl)methyl)acrylate (3k)

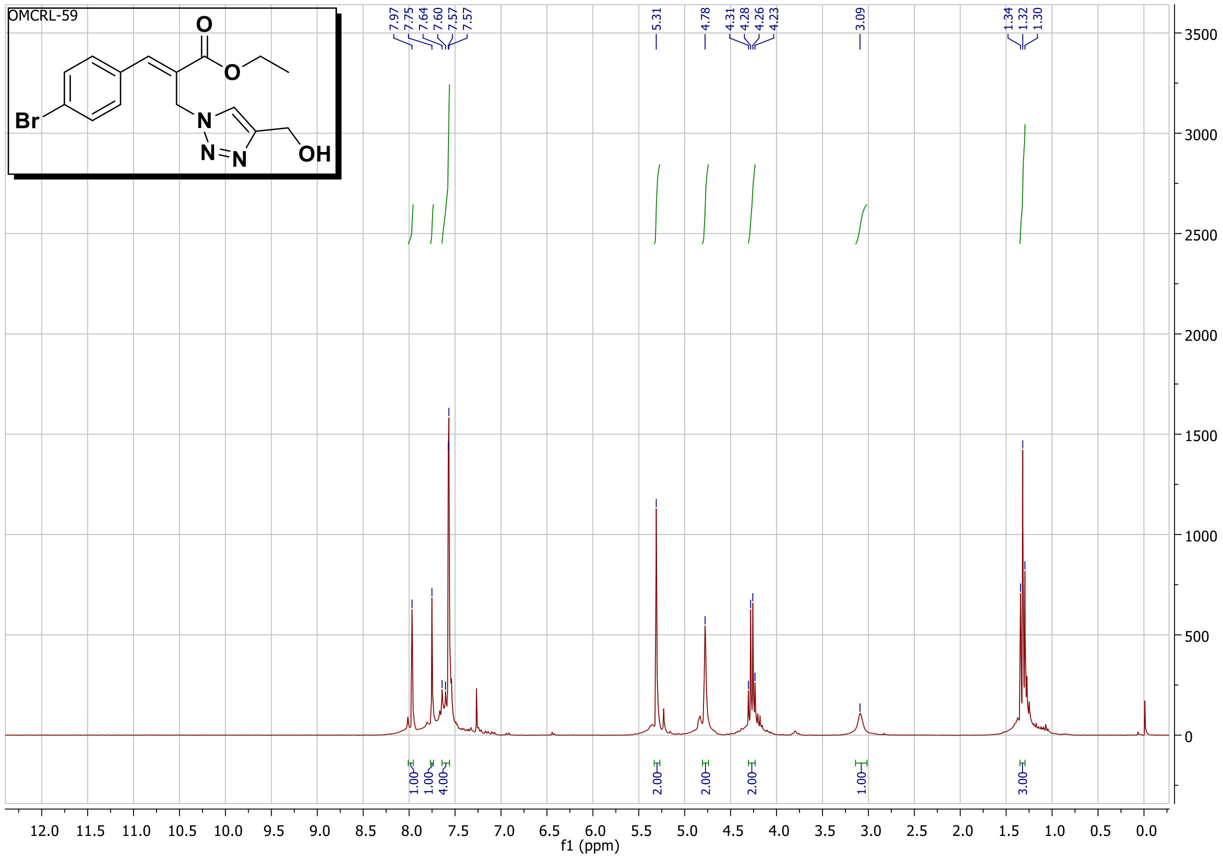

Figure S21 ^{1}H NMR spectrum of (*E*)-ethyl 3-(4-bromophenyl)-2-((4-(hydroxymethyl)-1*H*-1,2,3-triazol-1-yl)methyl)acrylate (**3k**)

Carbon NMR spectrum of (*E*)-ethyl 3-(4-bromophenyl)-2-((4-(hydroxymethyl)-1*H*-1,2,3-triazol-1-yl)methyl)acrylate (3k)

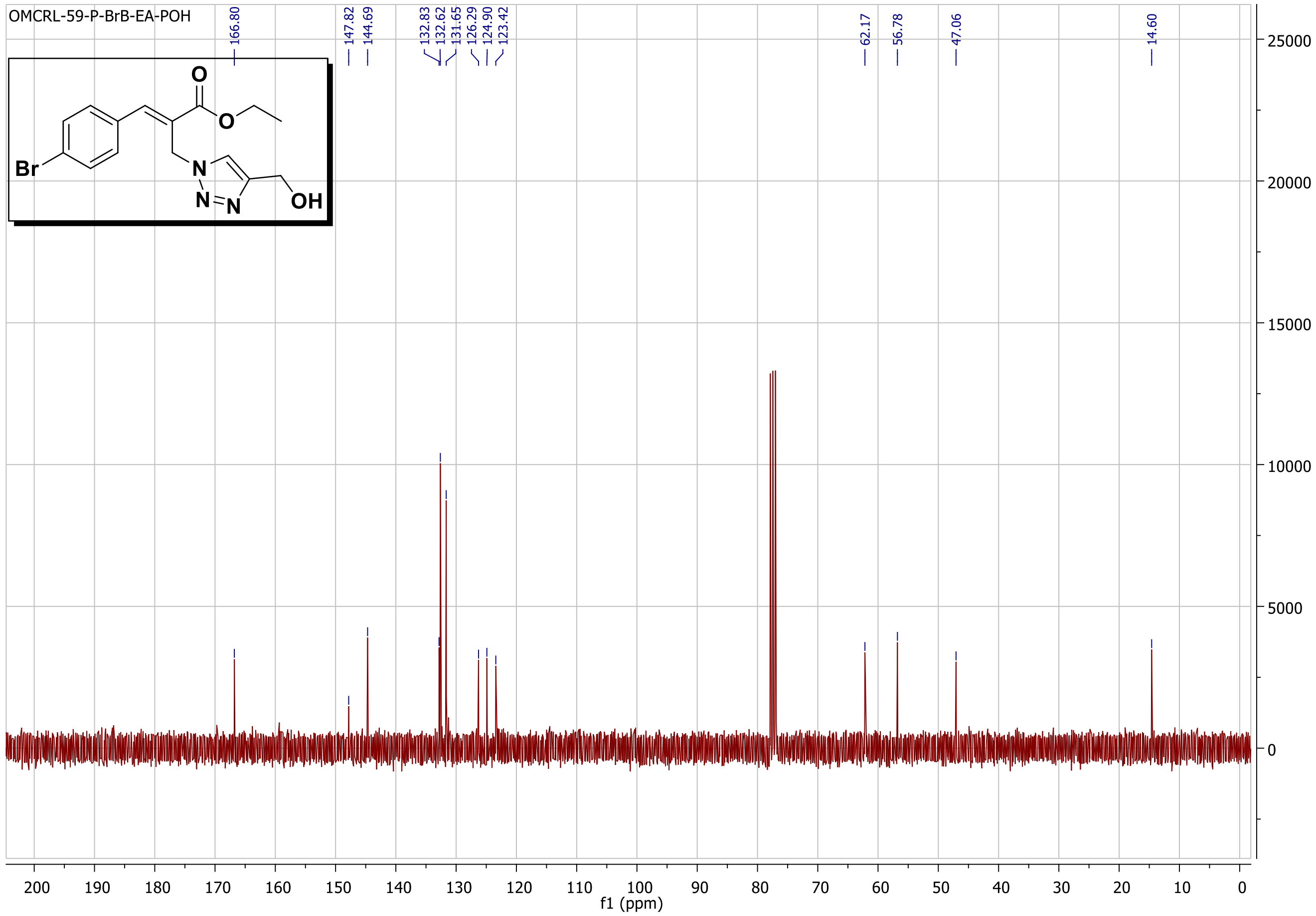

Figure S22 ^{13}C NMR spectrum of (*E*)-ethyl 3-(4-bromophenyl)-2-((4-(hydroxymethyl)-1*H*-1,2,3-triazol-1-yl)methyl)acrylate (**3k**)

Proton NMR spectrum of (*Z*)-3-(4-bromophenyl)-2-((4-(hydroxymethyl)-1*H*-1,2,3-triazol-1-yl)methyl)acrylonitrile (3l)

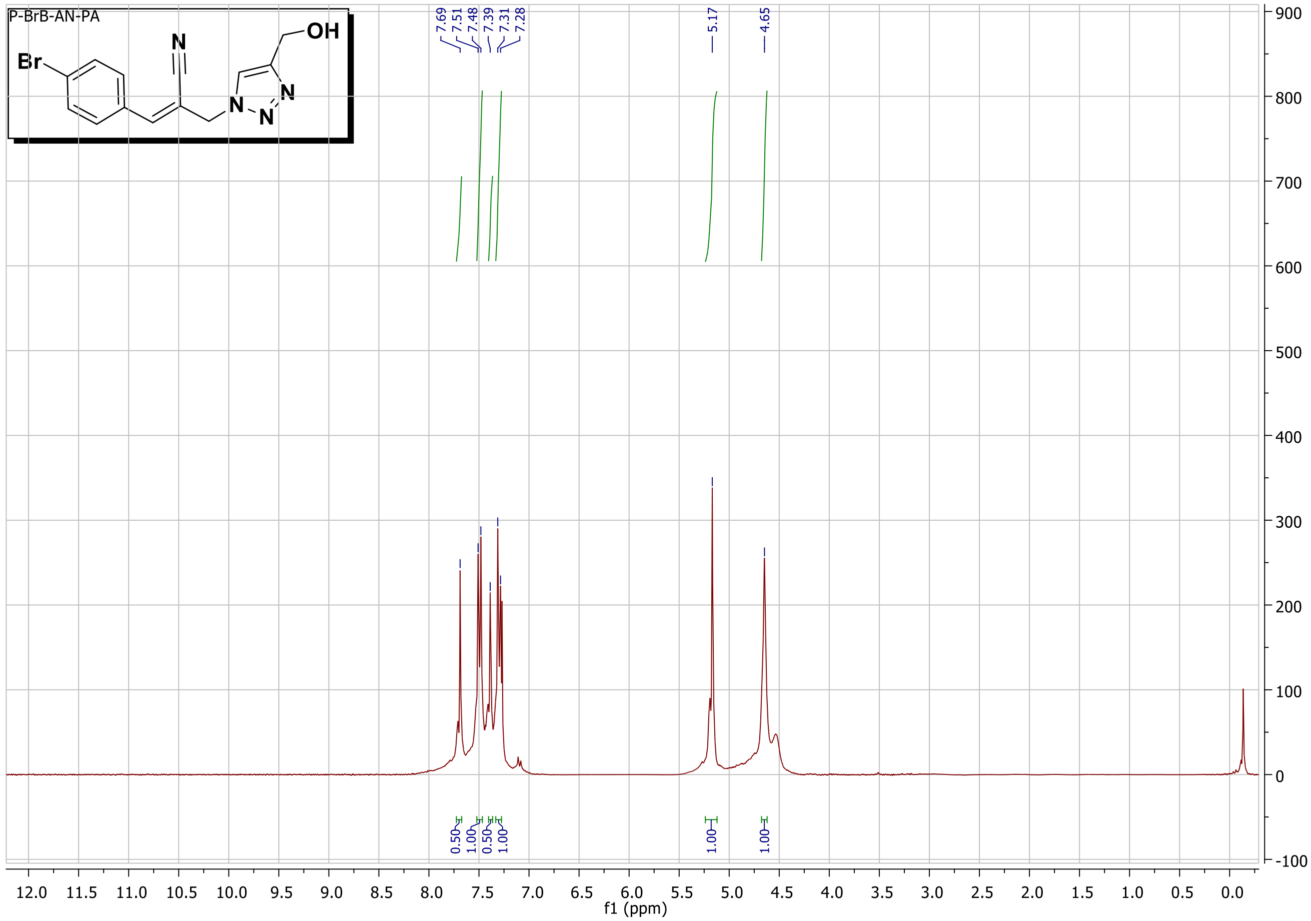

Figure S23 ^{1}H NMR spectrum of (*Z*)-3-(4-bromophenyl)-2-((4-(hydroxymethyl)-1*H*-1,2,3-triazol-1-yl)methyl)acrylonitrile (**3l**)

Carbon NMR spectrum of (*Z*)-3-(4-bromophenyl)-2-((4-(hydroxymethyl)-1*H*-1,2,3-triazol-1-yl)methyl)acrylonitrile (3l)

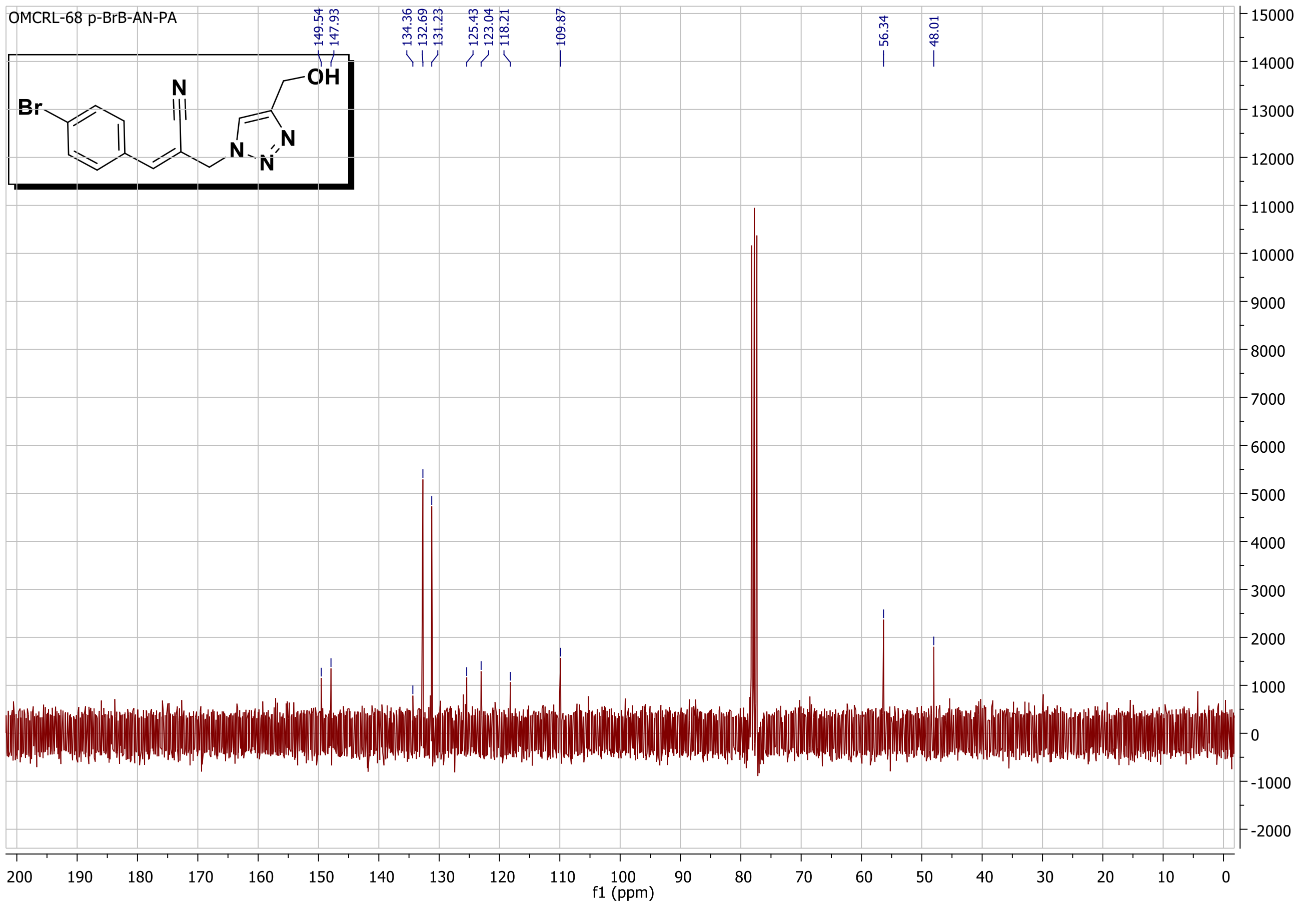

Figure S24 ^{13}C NMR spectrum of (*Z*)-3-(4-bromophenyl)-2-((4-(hydroxymethyl)-1*H*-1,2,3-triazol-1-yl)methyl)acrylonitrile (**3l**)

Proton NMR spectrum of (*E*)-methyl 2-((4-(hydroxymethyl)-1*H*-1,2,3-triazol-1-yl)methyl)-3-(4-nitrophenyl)acrylate (3m)

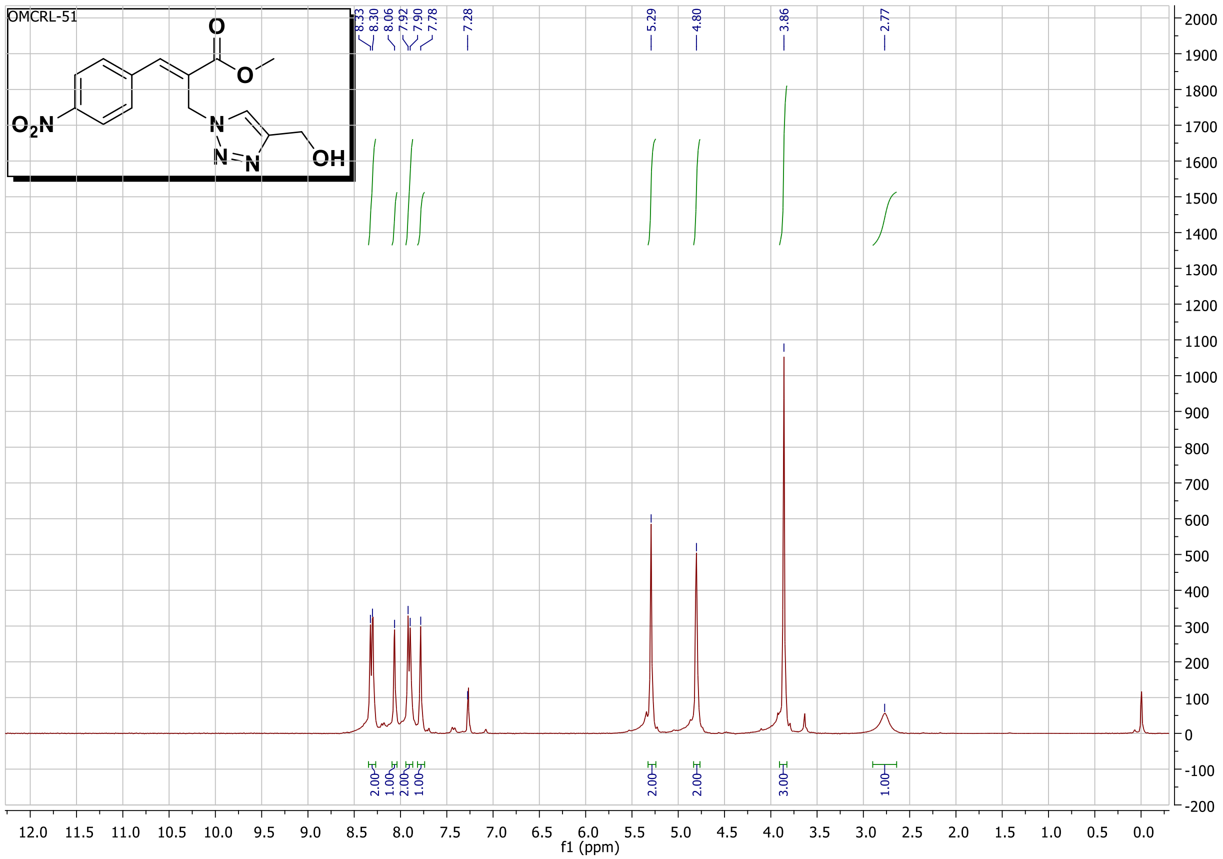

Figure S25 ^{1}H NMR spectrum of (*E*)-methyl 2-((4-(hydroxymethyl)-1*H*-1,2,3-triazol-1-yl)methyl)-3-(4-nitrophenyl)acrylate (**3m**)

Carbon NMR spectrum of (*E*)-methyl 2-((4-(hydroxymethyl)-1*H*-1,2,3-triazol-1-yl)methyl)-3-(4-nitrophenyl)acrylate (3m)

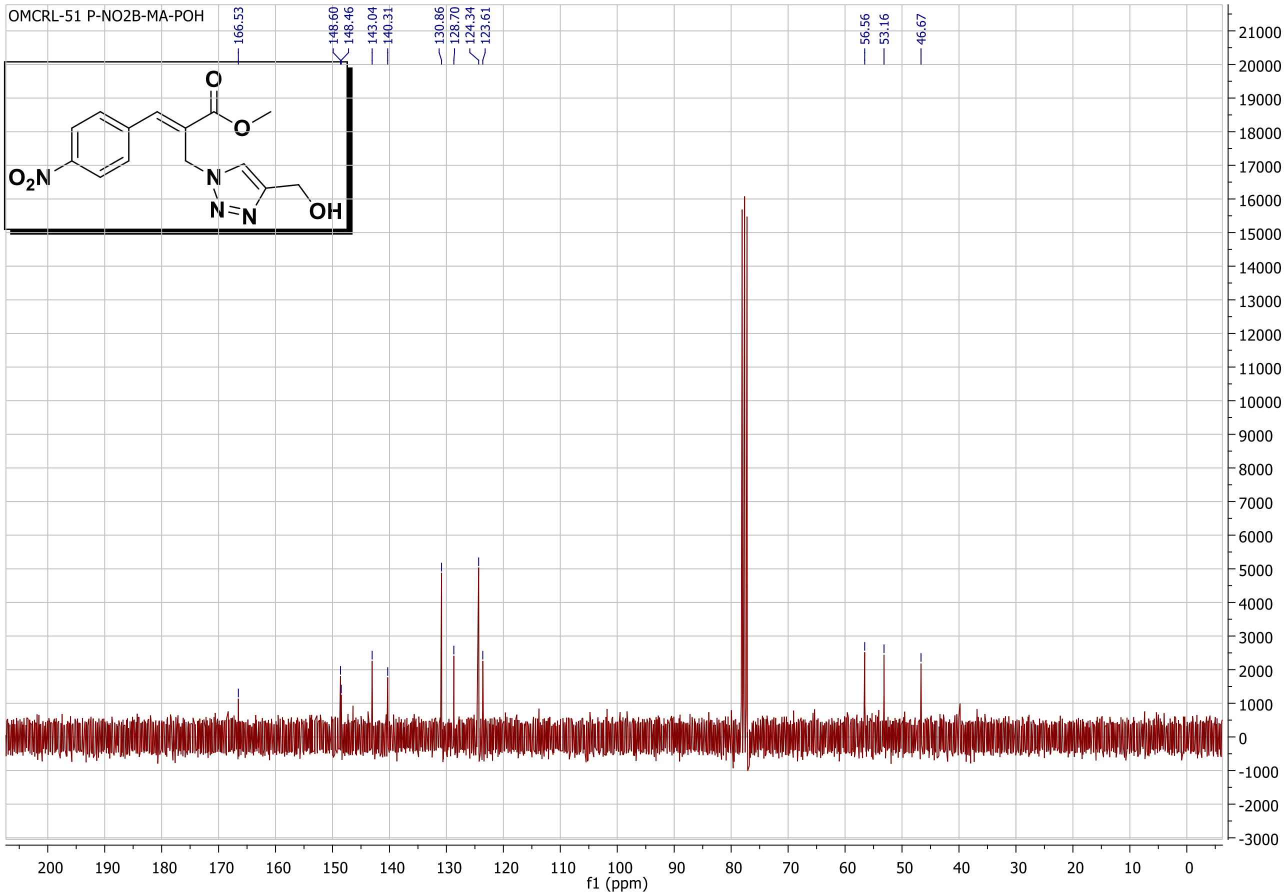

Figure S26 ^{13}C NMR spectrum of (*E*)-methyl 2-((4-(hydroxymethyl)-1*H*-1,2,3-triazol-1-yl)methyl)-3-(4-nitrophenyl)acrylate (**3m**)

Proton NMR spectrum of (*E*)-methyl 3-(furan-2-yl)-2-((4-phenyl-1*H*-1,2,3-triazol-1-yl)methyl)acrylate (3n)

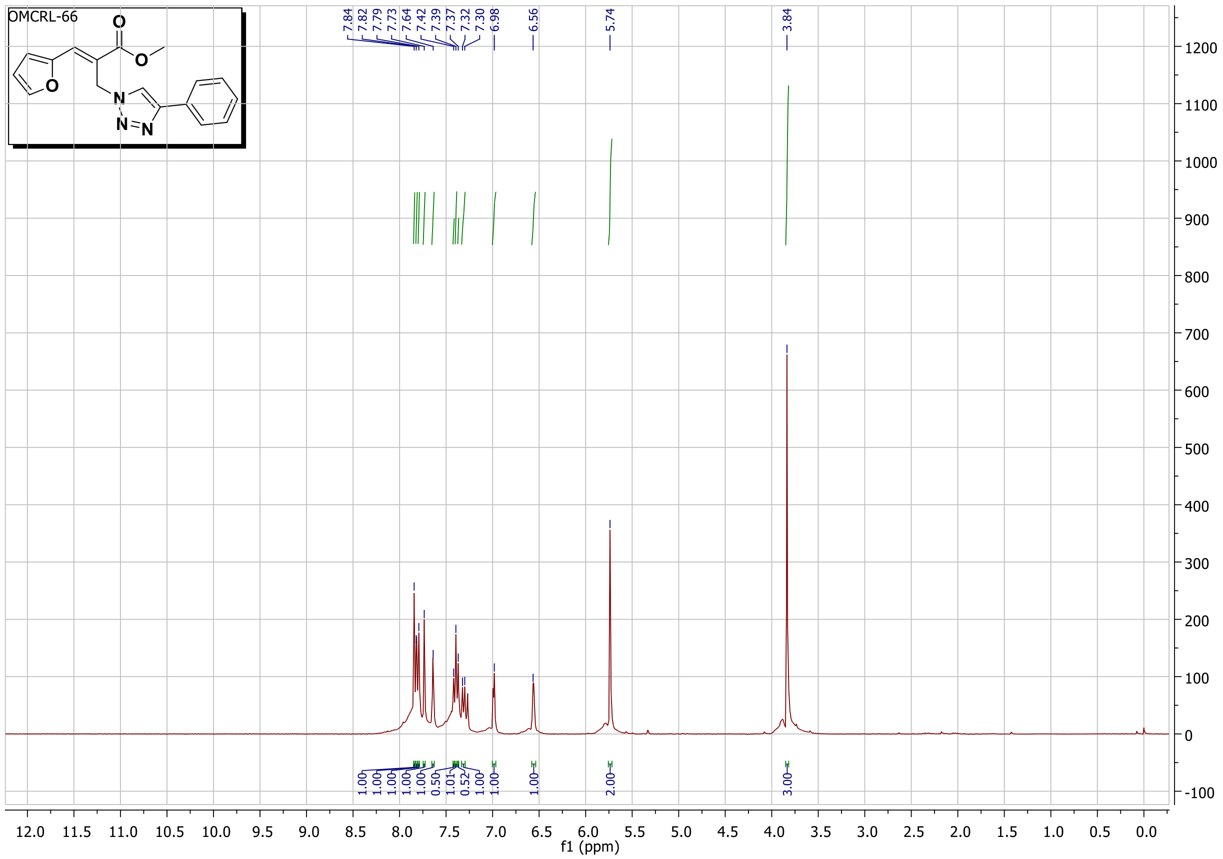

Figure S27 ^{1}H NMR spectrum of (*E*)-methyl 3-(furan-2-yl)-2-((4-phenyl-1*H*-1,2,3-triazol-1-yl)methyl)acrylate (**3n**)

Carbon NMR spectrum of (*E*)-methyl 3-(furan-2-yl)-2-((4-phenyl-1*H*-1,2,3-triazol-1-yl)methyl)acrylate (3n)

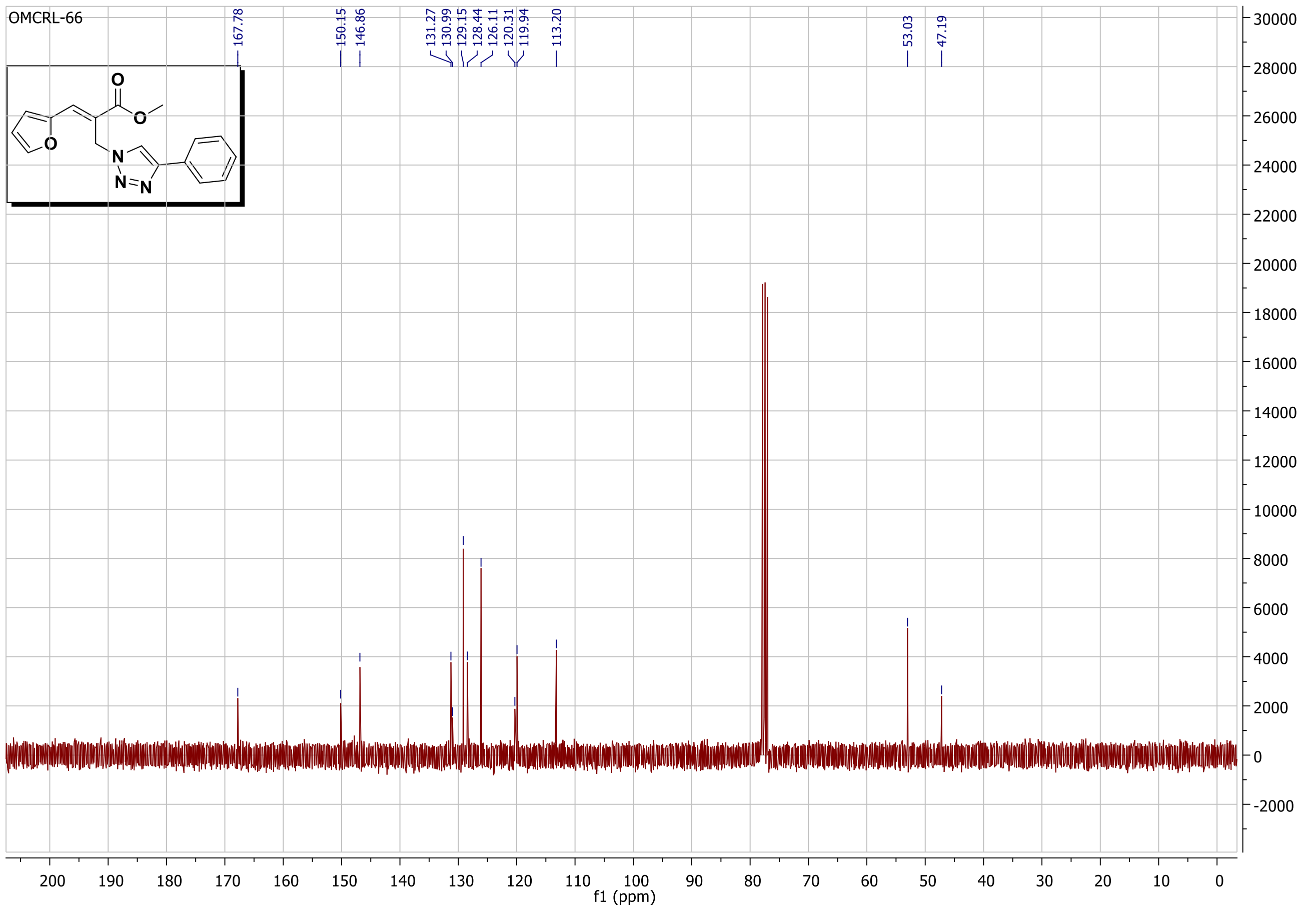

Figure S28 ^{13}C NMR spectrum of (*E*)-methyl 3-(furan-2-yl)-2-((4-phenyl-1*H*-1,2,3-triazol-1-yl)methyl)acrylate (**3n**)

Proton NMR spectrum of (*E*)-ethyl 3-(furan-2-yl)-2-((4-phenyl-1*H*-1,2,3-triazol-1-yl)methyl)acrylate (3o)

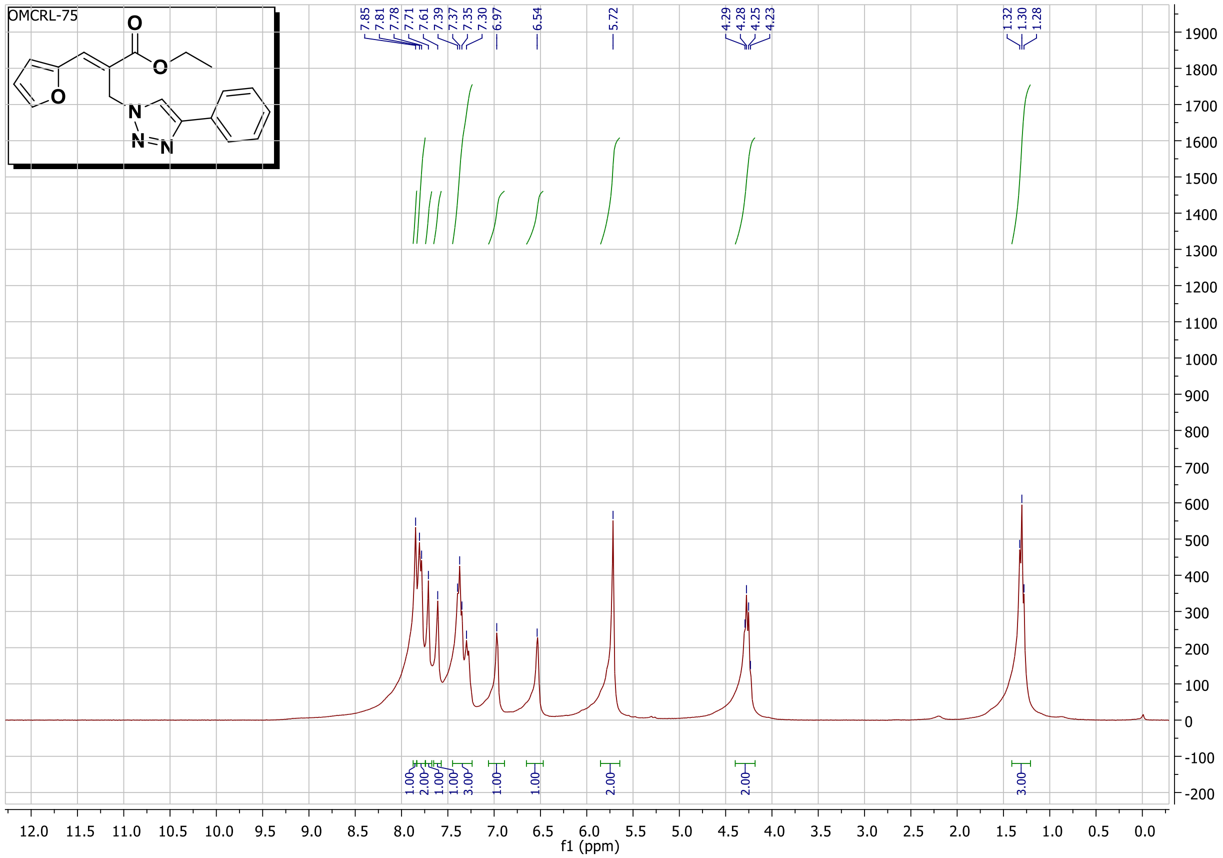

Figure S29 ^{1}H NMR spectrum of (*E*)-ethyl 3-(furan-2-yl)-2-((4-phenyl-1*H*-1,2,3-triazol-1-yl)methyl)acrylate (**3o**)

Carbon NMR spectrum of (*E*)-ethyl 3-(furan-2-yl)-2-((4-phenyl-1*H*-1,2,3-triazol-1-yl)methyl)acrylate (3o)

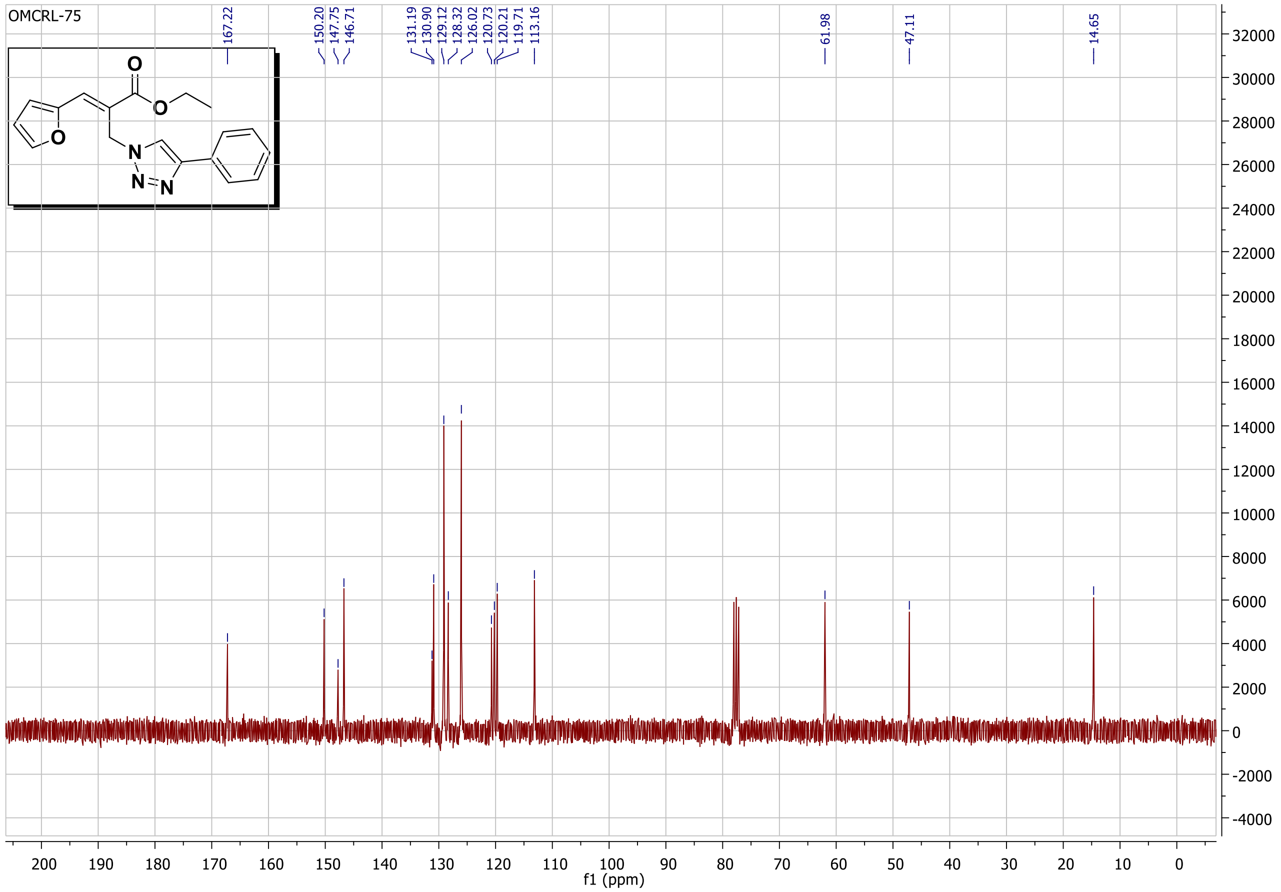

Figure S30 ^{13}C NMR spectrum of (*E*)-ethyl 3-(furan-2-yl)-2-((4-phenyl-1*H*-1,2,3-triazol-1-yl)methyl)acrylate (**3o**)

Proton NMR spectrum of (*E*)-methyl 2-((4-phenyl-1*H*-1,2,3-triazol-1-yl)methyl)-3-(thiophen-2-yl)acrylate (3p)

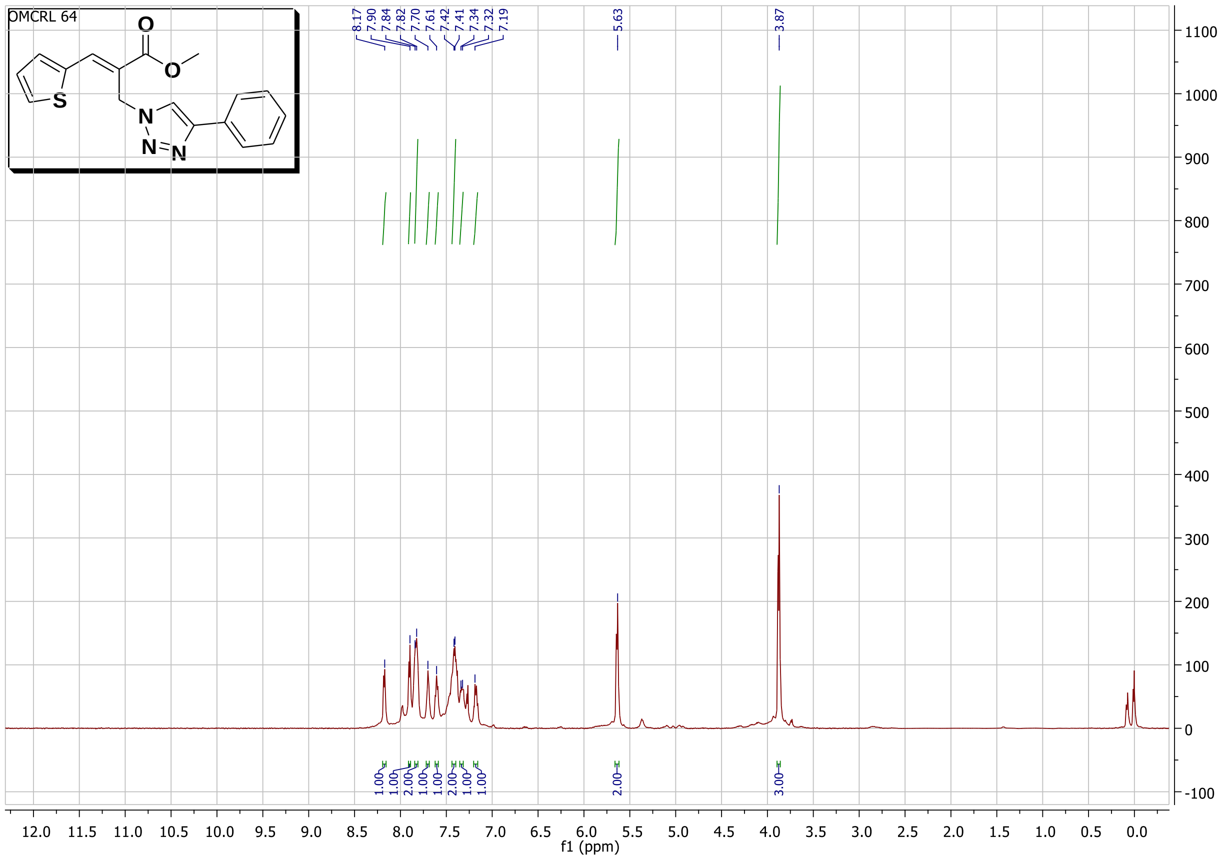

Figure S31 ^{1}H NMR spectrum of (*E*)-methyl 2-((4-phenyl-1*H*-1,2,3-triazol-1-yl)methyl)-3-(thiophen-2-yl)acrylate (**3p**)

Carbon NMR spectrum of (*E*)-methyl 2-((4-phenyl-1*H*-1,2,3-triazol-1-yl)methyl)-3-(thiophen-2-yl)acrylate (3p)

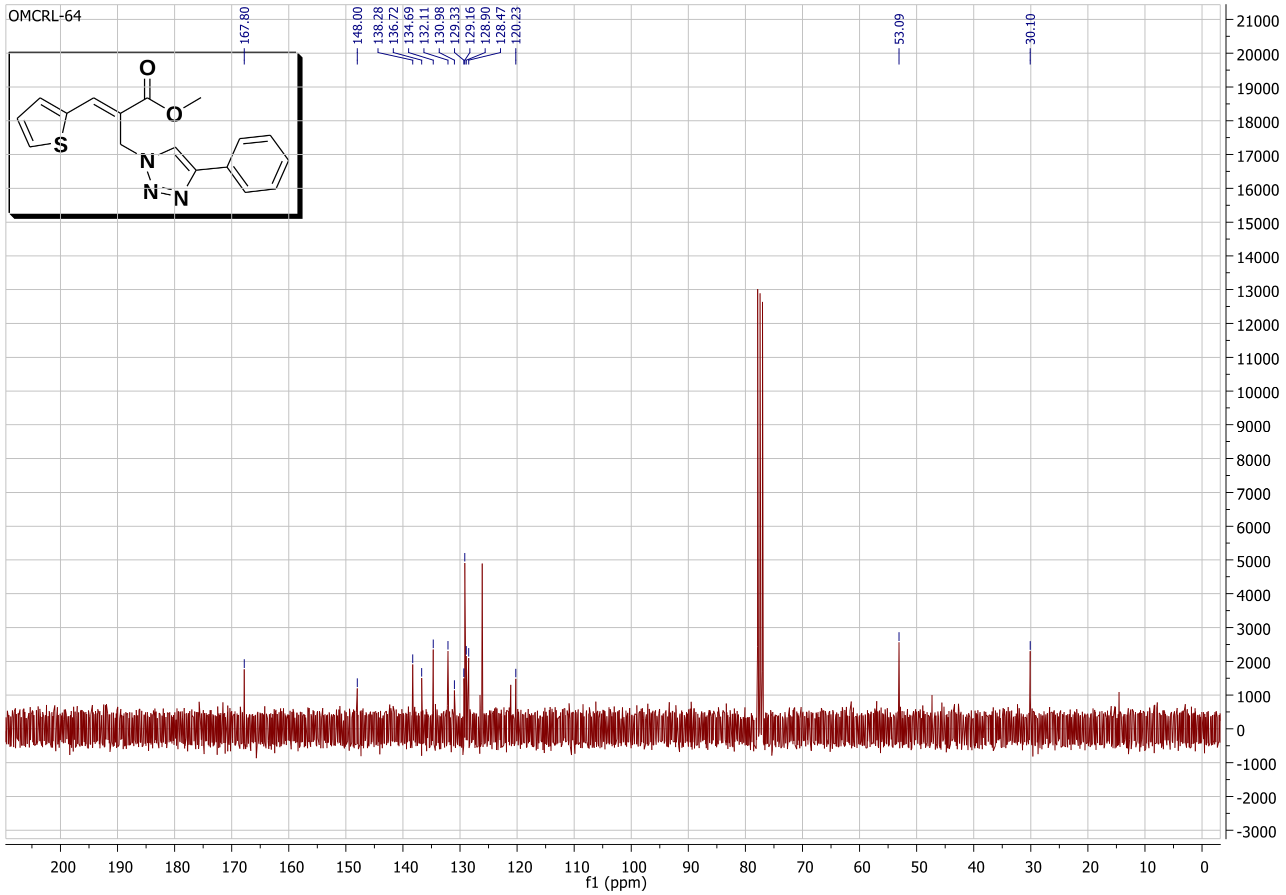

Figure S32 ^{13}C NMR spectrum of (*E*)-methyl 2-((4-phenyl-1*H*-1,2,3-triazol-1-yl)methyl)-3-(thiophen-2-yl)acrylate (**3p**)

Proton NMR spectrum of (*E*)-ethyl 2-((4-phenyl-1*H*-1,2,3-triazol-1-yl)methyl)-3-(thiophen-2-yl)acrylate (3q)

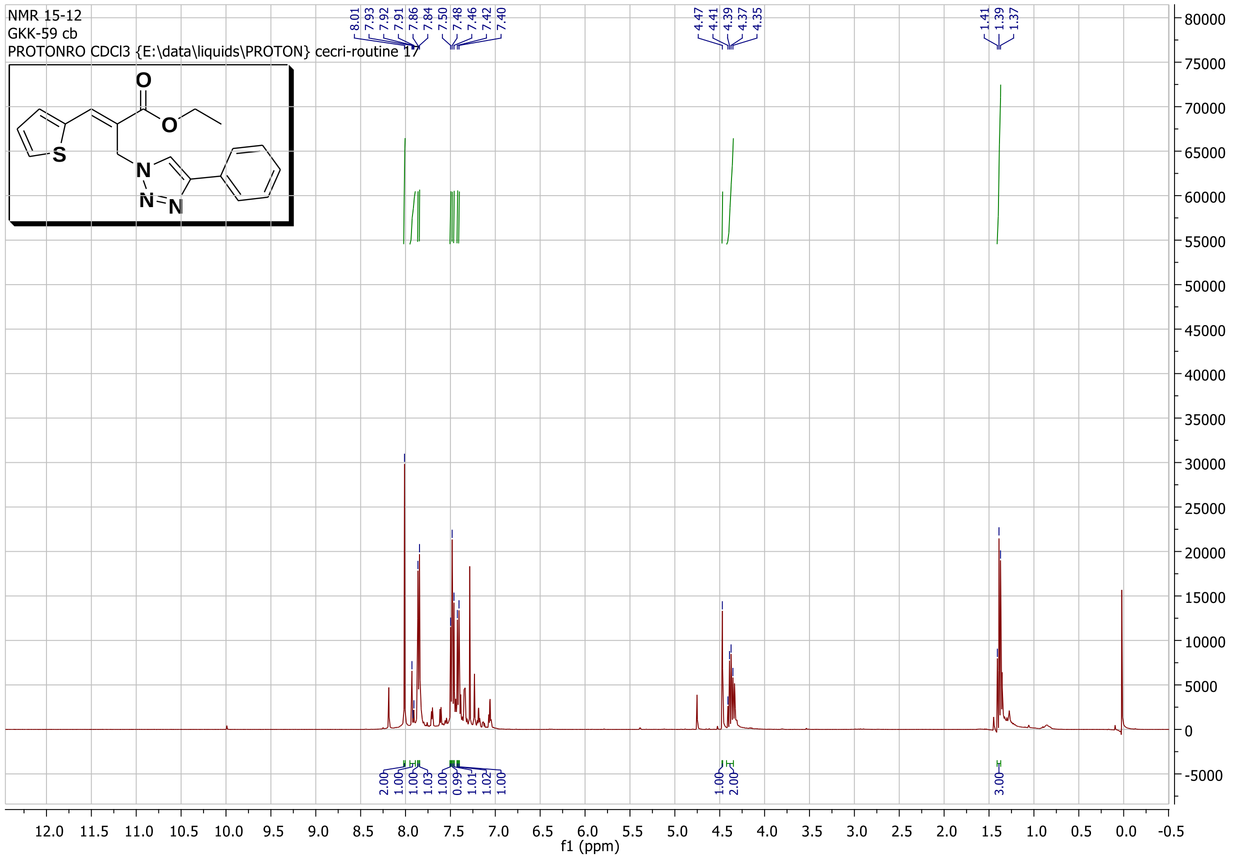

Figure S33 ^{1}H NMR spectrum of (*E*)-ethyl 2-((4-phenyl-1*H*-1,2,3-triazol-1-yl)methyl)-3-(thiophen-2-yl)acrylate (**3q**)

Carbon NMR spectrum of (*E*)-ethyl 2-((4-phenyl-1*H*-1,2,3-triazol-1-yl)methyl)-3-(thiophen-2-yl)acrylate (3q)

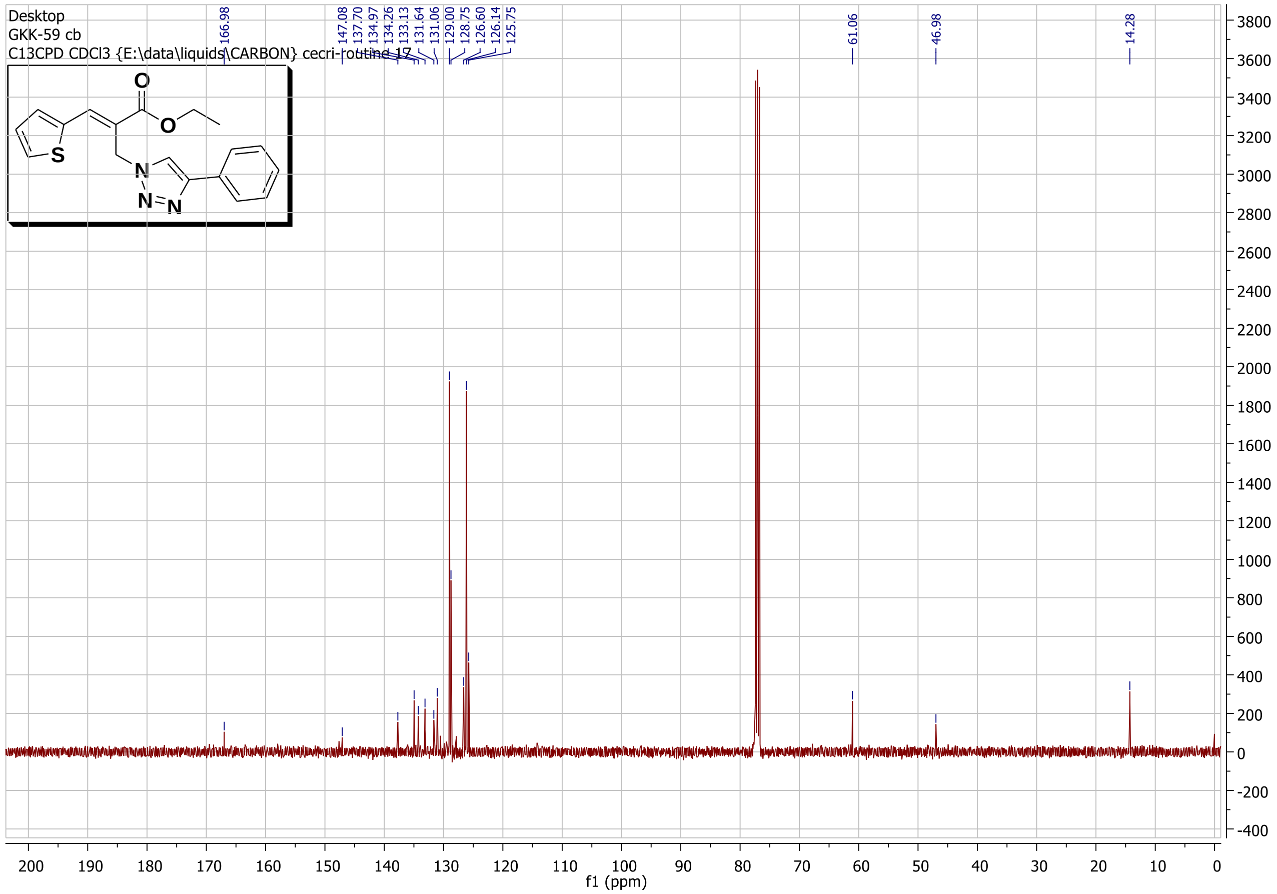

Figure S34 ^{13}C NMR spectrum of (*E*)-ethyl 2-((4-phenyl-1*H*-1,2,3-triazol-1-yl)methyl)-3-(thiophen-2-yl)acrylate (**3q**)

Copies of ^{1}H NMR and ^{13}C NMR spectra for compounds 4a–c

Proton NMR spectrum of 3-(bromomethyl)-2*H*-chromen-2-one (4a)

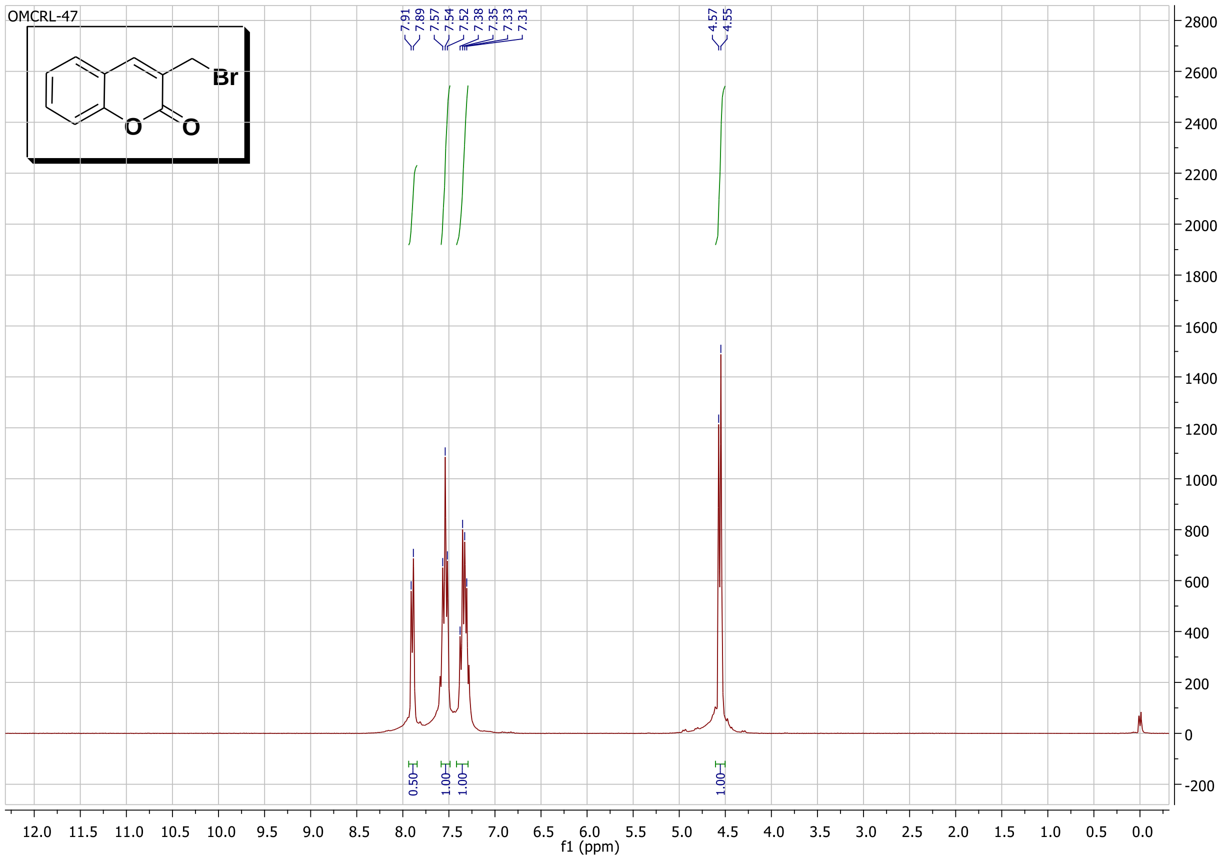

Figure S35 ^{1}H NMR spectrum of 3-(bromomethyl)-2*H*-chromen-2-one (**4a**)

Carbon NMR spectrum of 3-(bromomethyl)-2*H*-chromen-2-one (4a)

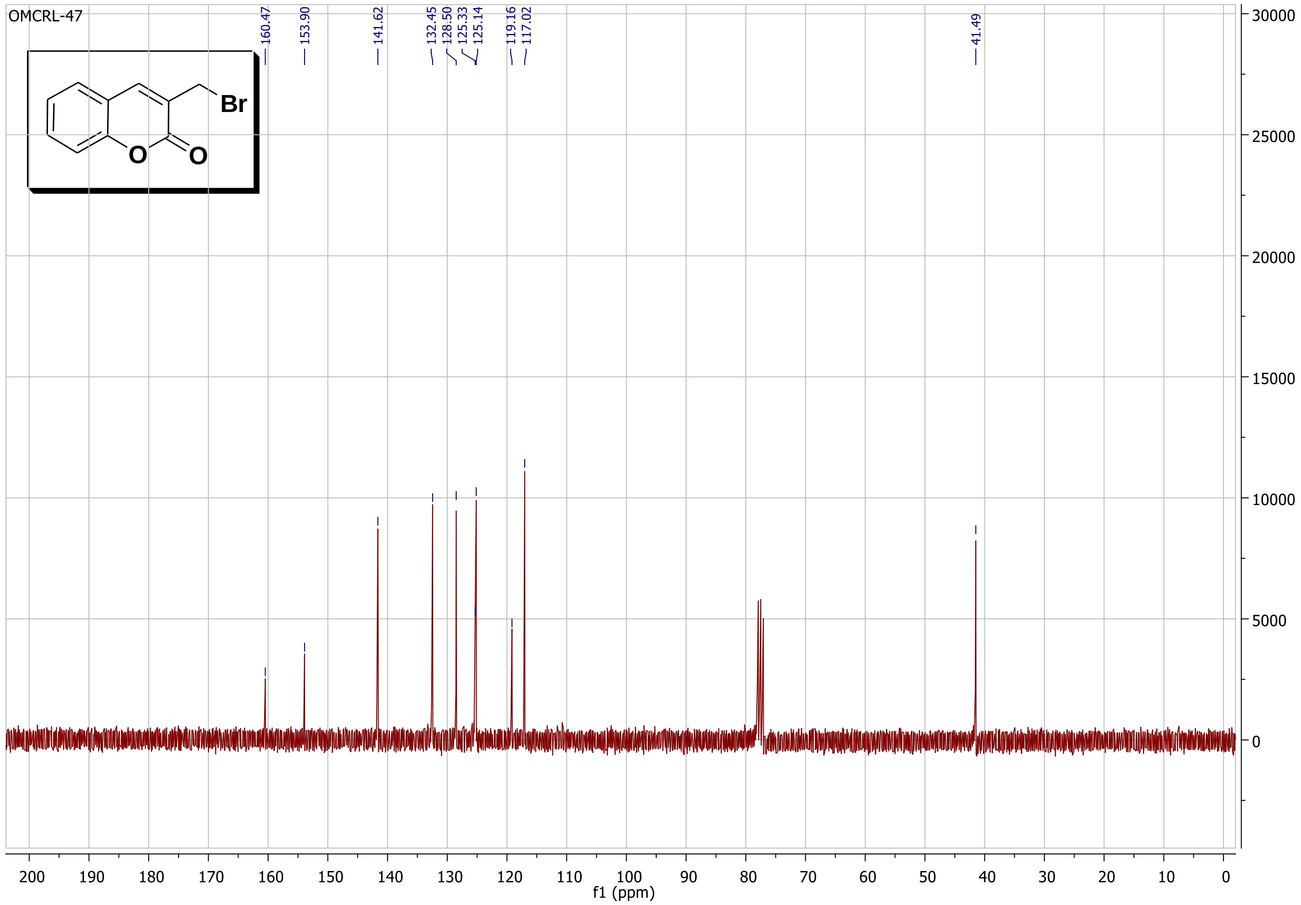

Figure S36 ^{13}C NMR spectrum of 3-(bromomethyl)-2*H*-chromen-2-one (**4a**)

Proton NMR spectrum of 3-(bromomethyl)-8-methoxy-2*H*-chromen-2-one (4b)

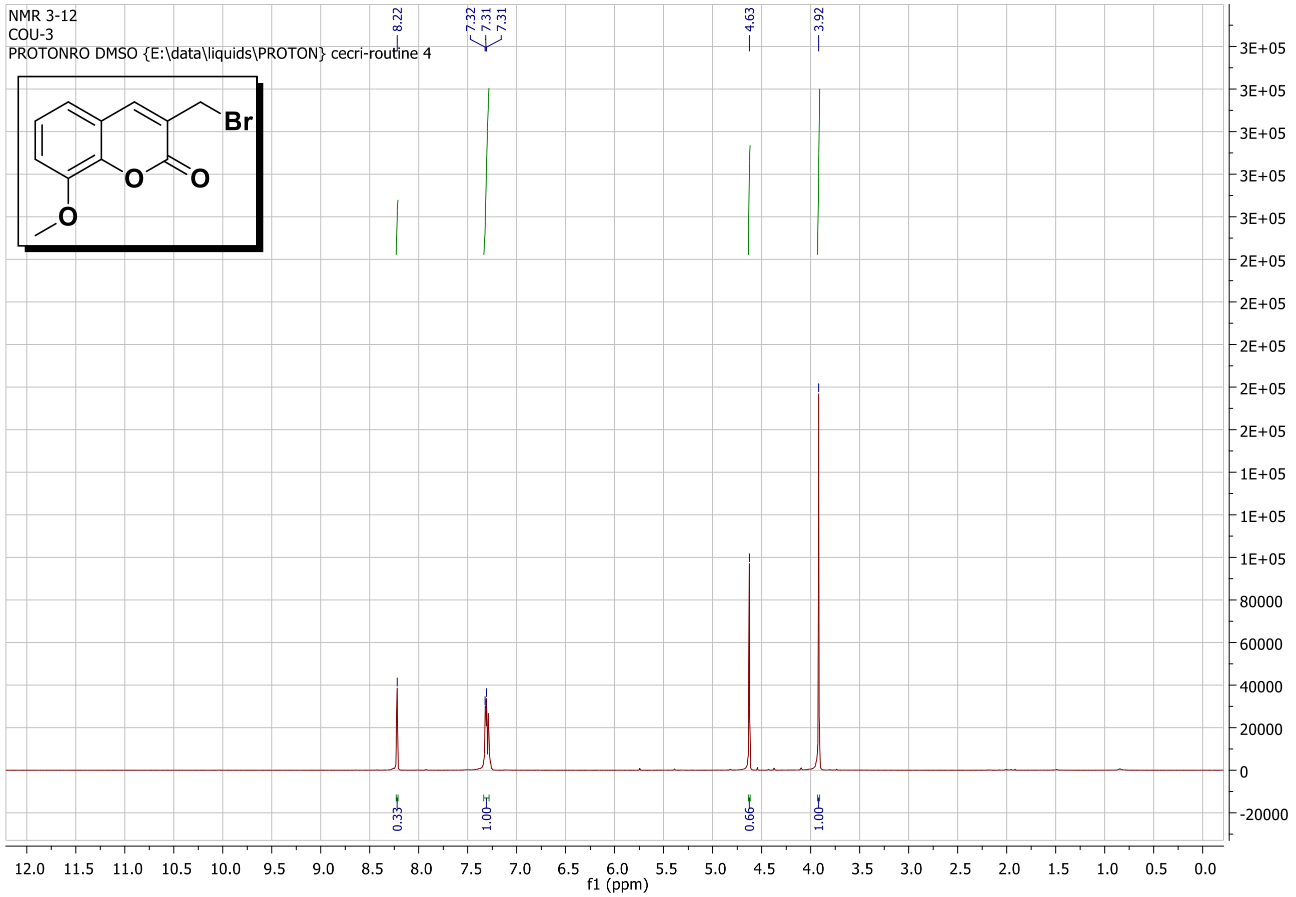

Figure S37 ^{1}H NMR spectrum of 3-(bromomethyl)-8-methoxy-2*H*-chromen-2-one (**4b**)

Carbon NMR spectrum of 3-(bromomethyl)-8-methoxy-2*H*-chromen-2-one (4b)

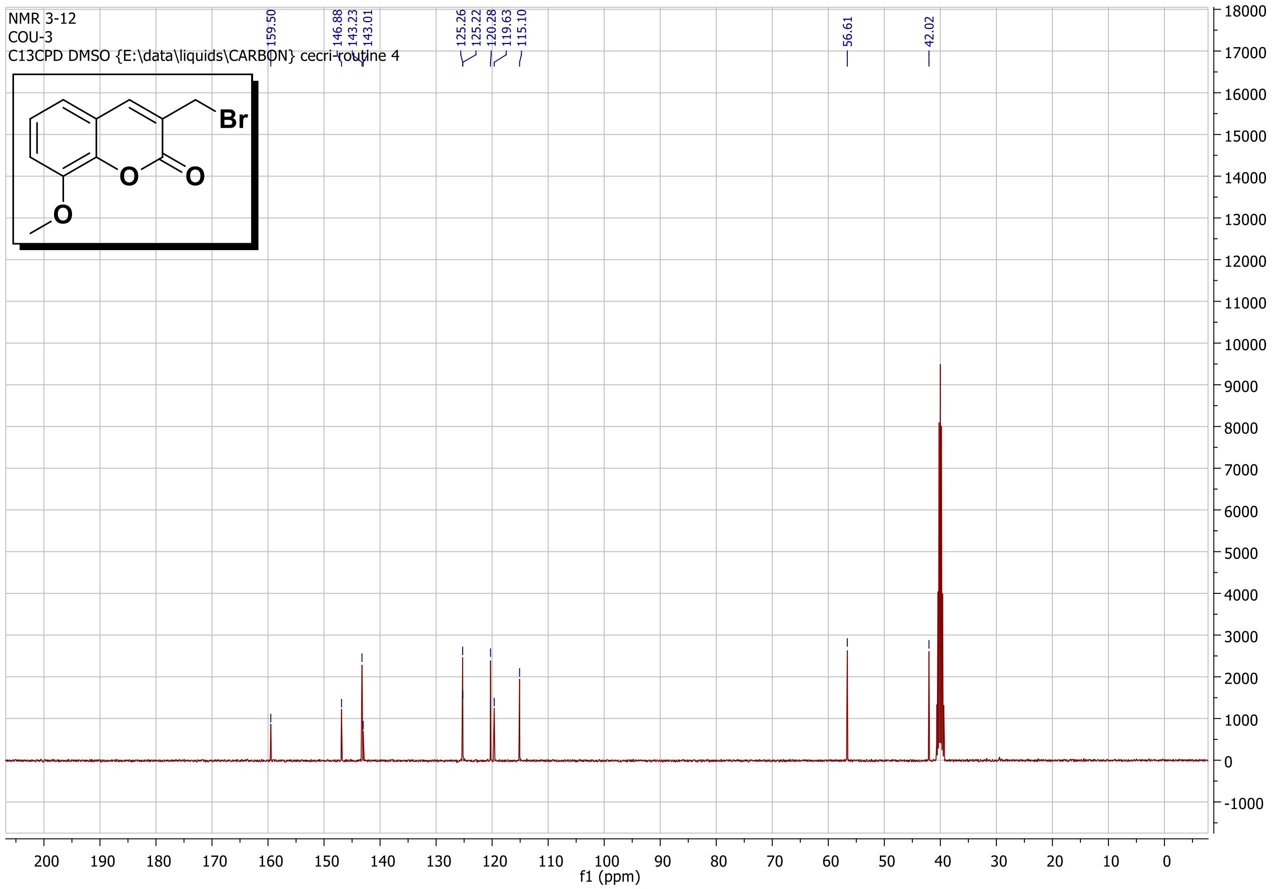

Figure S38 ^{13}C NMR spectrum of 3-(bromomethyl)-8-methoxy-2*H*-chromen-2-one (**4b**)

Proton NMR spectrum of 6-bromo-3-(bromomethyl)-2*H*-chromen-2-one (4c)

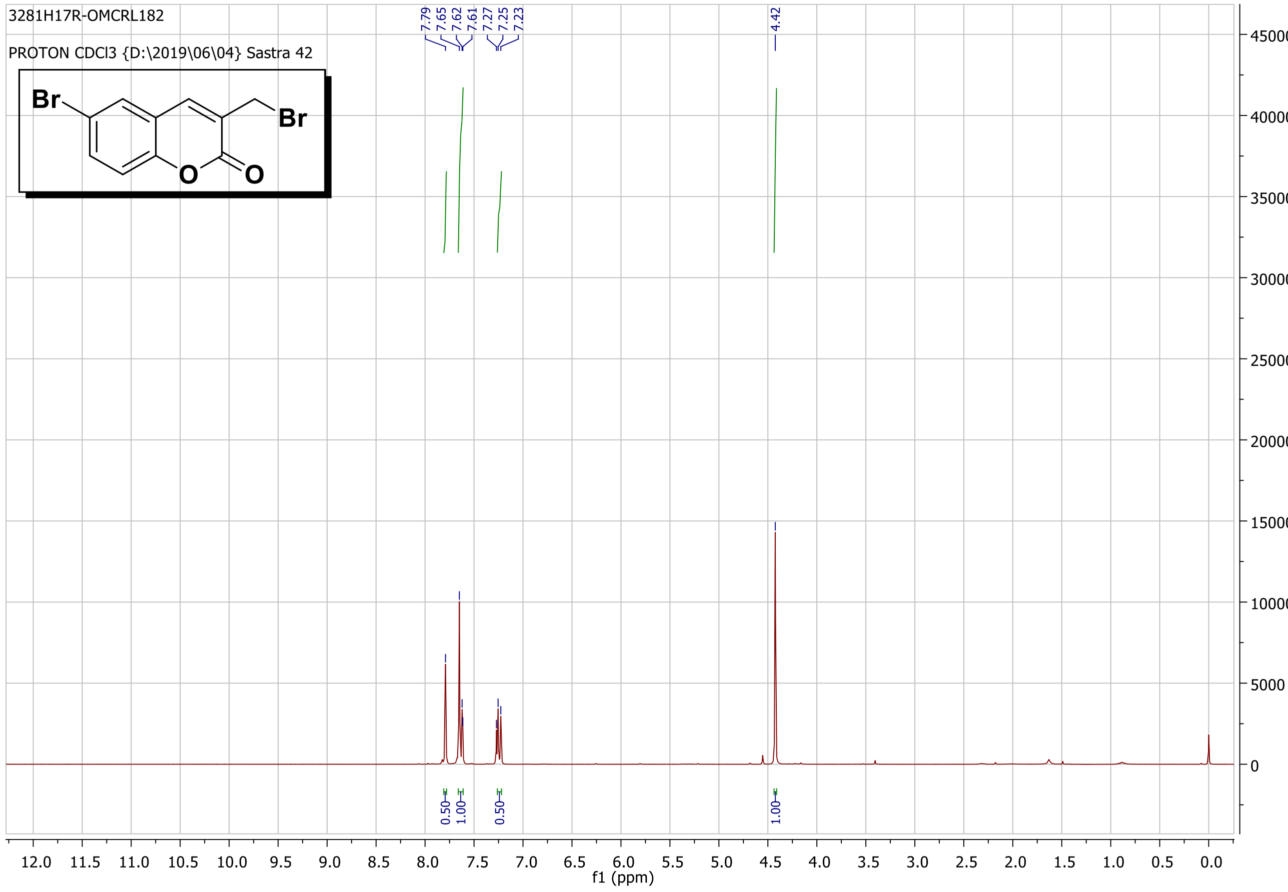

Figure S39 ^{1}H NMR spectrum of 6-bromo-3-(bromomethyl)-2*H*-chromen-2-one (**4c**)

Carbon NMR spectrum of 6-bromo-3-(bromomethyl)-2*H*-chromen-2-one (4c)

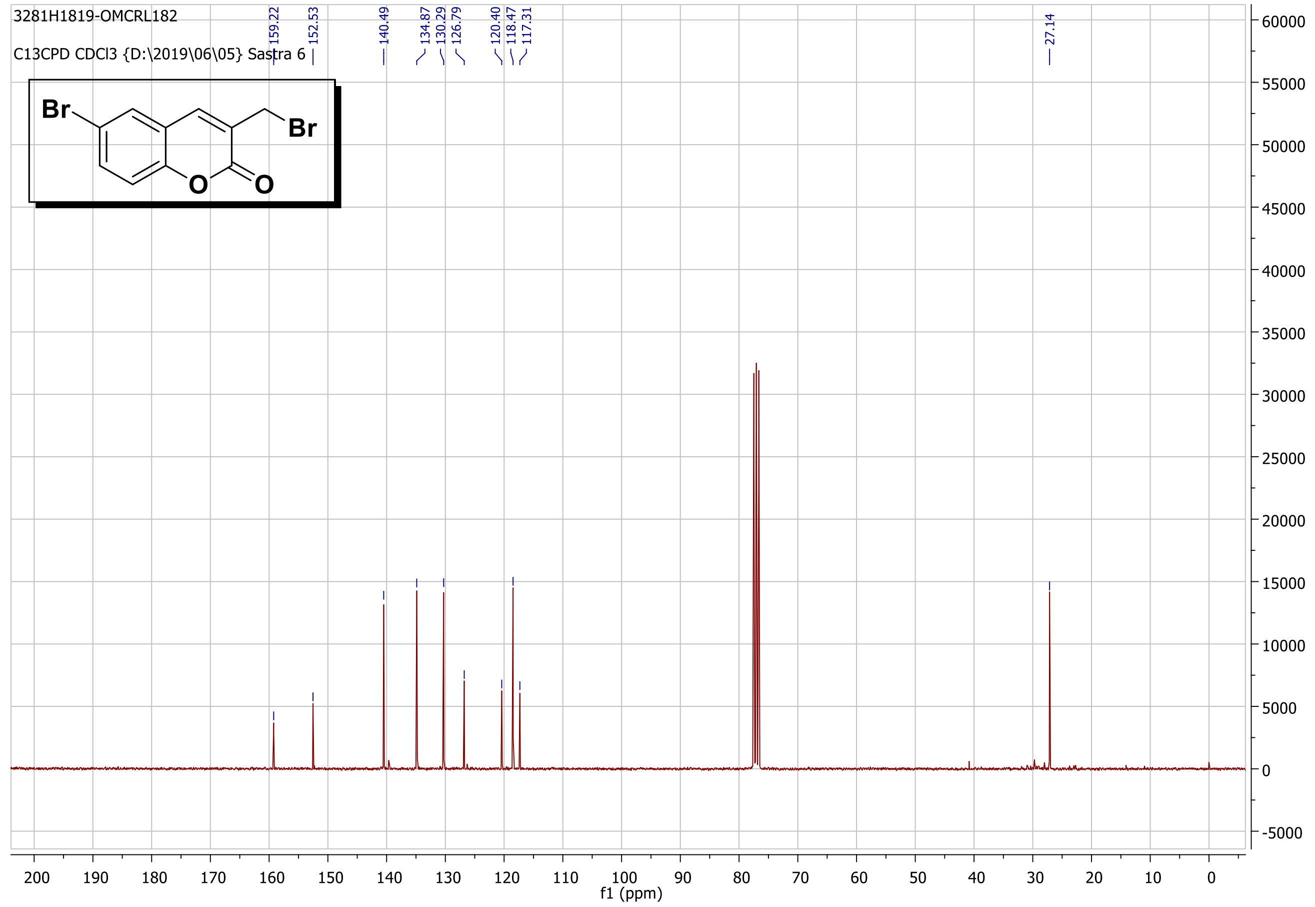

Figure S40 ^{13}C NMR spectrum of 6-bromo-3-(bromomethyl)-2*H*-chromen-2-one (**4c**)

Spectral data of (*E*)/(*Z*)-cinnamyl-1*H*-triazoles 3a–q

(*E*)-Methyl 2-((4-hydroxymethyl)-1*H*-1,2,3-triazol-1-yl)methyl)-3-phenylacrylate (3a)

Yield: 295 mg (83%); white solid; mp 92-94 °C.

^{1}H NMR ($CDCl_3$, 300 MHz): δ = 3.83 (s, 3H, estr-CH_3), 4.78 (s, 2H, Trz-CH_2), 5.37 (s, 2H, Trz-CH_2OH), 7.43-7.45 (m, 3H, Aro-H), 7.61-7.64 (m, 2H, Aro-H), 7.74 (s, 1H, Trz-CH), 8.08 (s, 1H, Alkene-CH).

^{13}C NMR ($CDCl_3$, 300 MHz): δ = 47.23, 52.98, 56.75, 123.15, 125.30, 129.39, 130.07, 130.43, 133.91, 146.44, 147.84, 167.52.

HRMS: m/z Calcd for $C_{14}H_{15}N_3O_3$ $[M+H]^+$: 274.1113; found: 274.1136.

(*E*)-Methyl 3-phenyl-2-((4-phenyl-1*H*-1,2,3-triazol-1-yl)methyl)acrylate (3b) [2]

Yield: 365 mg (88%); yellowish white solid; mp 114-116 °C.

^{1}H NMR ($CDCl_3$, 300 MHz): δ = 3.84 (s, 3H, estr-CH_3), 5.42 (s, 2H, Trz-CH_2), 7.38-7.48 (m, 5H, Aro-H), 7.68-7.85 (m, 5H, Aro-H), 7.98 (s, 1H, Trz-CH), 8.10 (s, 1H, Alkene-CH).

^{13}C NMR ($CDCl_3$, 300 MHz): δ = 47.34, 53.02, 121.20, 125.39, 126.14, 126.50, 128.54, 129.22, 129.33, 129.41, 130.18, 130.46, 133.95, 146.55, 147.92, 167.69.

HRMS: m/z Calcd for $C_{19}H_{17}N_3O_2$ $[M+H]^+$: 320.1321; found: 320.1311.

(*E*)-Ethyl 2-((4-hydroxymethyl)-1*H*-1,2,3-triazol-1-yl)methyl)-3-phenylacrylate (3c)

Yield: 278 mg (80%); white solid; mp 81-83 °C.

^{1}H NMR ($CDCl_3$, 300 MHz): δ = 1.30-1.34 (t, *J* = 6 Hz, 3H, estr-CH_3), 3.50 (s, 1H, CH_2-OH), 4.24-4.31 (q, *J* = 6Hz, 2H, estr-CH_2), 4.78 (s, 2H, Trz-CH_2), 5.36 (s, 2H, Trz-CH_2OH), 7.41-7.61 (m, 5H, Aro-H), 7.74 (s, 1H, Trz-CH), 8.07 (s, 1H, Alkene-CH).

^{13}C NMR ($CDCl_3$, 300 MHz): δ = 14.59, 47.22, 56.73, 62.03, 123.14, 125.64, 129.35, 130.04, 130.32, 133.99, 146.09, 147.86, 167.02.

HRMS: m/z Calcd for $C_{15}H_{17}N_3O_3$ $[M+H]^+$: 288.1270; found: 288.1258.

(*E*)-Ethyl 3-phenyl-2-((4-phenyl-1*H*-1,2,3-triazol-1-yl)methyl)acrylate (3d) [1]

Yield: 331 mg (82%); white crystals; mp 100-102 °C.

^{1}H NMR ($CDCl_3$, 300 MHz): δ = 1.29-1.34 (t, *J* = 6 Hz, 3H, estr-CH_3), 4.24-4.31 (q, *J* = 6 Hz, 2H, estr-CH_2), 5.40 (s, 2H, Trz-CH_2), 7.30-7.85 (m, 10H, Aro-H), 7.98 (s, 1H, Trz-CH), 8.08 (s, 1H, Alkene-CH).

^{13}C NMR ($CDCl_3$, 300 MHz): δ = 14.60, 47.27, 61.95, 121.10, 125.92, 126.09, 128.40, 129.16, 129.33, 130.10, 130.27, 131.19, 134.12, 145.99, 147.82, 167.04.

HRMS: m/z Calcd for $C_{20}H_{19}N_3O_2$ $[M+H]^+$: 334.1477; found: 334.1453.

(*Z*)-2-((4-(Hydroxymethyl)-1*H*-1,2,3-triazol-1-yl)methyl)-3-phenylacrylonitrile (3e) [2]

Yield: 339 mg (90%); yellowish solid; mp 103-105 °C.

^{1}H NMR ($CDCl_3$, 300 MHz): δ = 2.86 (s, 1H, Trz-CH_2-OH), 4.53 (s, 2H, Trz-CH_2), 5.09 (s, 2H, Trz-CH_2-OH), 7.15 (s, 1H, Alkene-CH), 7.22-7.55 (m, 5H, Aro-H), 7.64 (s, 1H, Trz-CH).

^{13}C NMR ($CDCl_3$, 300 MHz): δ = 58.51, 61.15, 110.21, 122.11, 127.67, 134.16, 134.36, 136.57, 137.46, 153.20, 154.41.

HRMS: m/z Calcd for $C_{13}H_{12}N_4O$ $[M+H]^+$: 241.1011; found: 241.1023.

(*Z*)-3-Phenyl-2-((4-phenyl-1*H*-1,2,3-triazol-1-yl)methyl)acrylonitrile (3f) [2]

Yield: 413 mg (92%); white crystals; mp 128-130 °C.

^{1}H NMR ($CDCl_3$, 300 MHz): δ = 5.30 (s, 2H, Trz-CH_2), 7.33-7.84 (m, 10H, Aro-H), 7.87 (s, 1H, Alkene-CH), 7.98 (s, 1H, Trz-CH).

^{13}C NMR ($CDCl_3$, 300 MHz): δ = 54.03, 104.94, 117.41, 120.28, 126.25, 128.88, 129.32, 129.49, 129.71, 130.52, 132.04, 132.48, 148.56, 149.01.

HRMS: m/z Calcd for $C_{18}H_{14}N_4$ $[M+H]^+$: 287.1218; found: 287.1209.

(*E*)-Methyl 3-(4-chlorophenyl)-2-((4-(hydroxymethyl)-1*H*-1,2,3-triazole-1-yl)methyl)acrylate (3g)

Yield: 244 mg (72%); white solid; mp 125-127 °C.

^{1}H NMR ($CDCl_3$, 300 MHz): δ = 3.38 (s, 1H, Trz-CH_2OH), 3.82 (s, 3H, estr-CH_3), 4.77 (s, 2H, Trz-CH_2), 5.31 (s, 2H, Trz-CH_2OH), 7.40-7.43 (d, *J* = 9 Hz, 2H, Aro-H), 7.61-7.63 (d, *J* = 6 Hz, 2H, Aro-H), 7.75 (s, 1H, Trz-CH), 7.99 (s, 1H, Alkene-CH).

^{13}C NMR ($CDCl_3$, 300 MHz): δ = 47.03, 52.99, 56.74, 123.32, 125.95, 129.65, 131.45, 132.33, 136.60, 144.88, 167.27.

HRMS: m/z Calcd for $C_{14}H_{14}ClN_3O_3$ $[M+H]^+$: 308.0724; found: 308.0719.

(*E*)-Methyl 3-(4-chlorophenyl)-2-(4-phenyl-1*H*-1,2,3-triazole-1-yl)methyl)acrylate (3h) [2]

Yield: 304 mg (78%); white solid; mp 141-143 °C.

^{1}H NMR ($CDCl_3$, 300 MHz): δ = 3.76 (s, 3H, estr-CH_3), 5.29 (s, 2H, Trz-CH_2), 7.33-7.38 (m, 5H, Aro-H), 7.62-7.65 (d, *J*= 9Hz, 2H, Aro-H), 7.80-7.82 (d, *J*= 6Hz, 2H, Aro-H), 7.93 (s, 1H, Trz-CH), 8.01 (s, 1H, Alkene-CH).

^{13}C NMR ($CDCl_3$, 300 MHz): δ = 47.11, 53.00, 121.67, 125.95, 126.05, 128.53, 129.22, 129.56, 130.92, 131.60, 132.40, 136.41, 144.77, 147.76, 167.29.

HRMS: m/z Calcd for $C_{19}H_{16}ClN_3O_2$ $[M+H]^+$: 354.0931; found: 354.0928.

(*E*)-Methyl 3-(4-bromophenyl)-2-((4-(hydroxymethyl)-1*H*-1,2,3-triazol-1-yl)methyl)acrylate (3i)

Yield: 259 mg (80%); white solid; mp 116-118 °C.

^{1}H NMR ($CDCl_3$, 300 MHz): δ = 3.83 (s, 3H, estr-CH_3), 4.79 (s, 2H, Trz-CH_2), 5.32 (s, 2H, Trz-CH_2OH), 7.58 (s, 4H, Aro-H), 7.77 (s, 1H, Trz-CH), 7.98 (s, 1H, Alkene-CH).

^{13}C NMR ($CDCl_3$, 300 MHz): δ = 47.05, 53.07, 56.63, 123.46, 124.98, 125.98, 131.66, 132.64, 132.75, 144.99, 147.82, 167.28.

HRMS: m/z Calcd for $C_{14}H_{14}BrN_3O_3$ $[M+H]^+$: 352.0219; found: 352.0210.

(*E*)-Methyl 3-(4-bromophenyl)-2-((4-phenyl-1*H*-1,2,3-triazol-1-yl)methyl)acrylate (3j)

Yield: 308 mg (84%); yellow solid; mp 132-134 °C.

^{1}H NMR ($CDCl_3$, 300 MHz): δ = 3.85 (s, 3H, estr-CH_3), 5.38 (s, 2H, Trz-CH_2), 7.34-7.86 (m, 9H, Aro-H), 8.01 (s, 1H, Trz-CH), 8.02 (s, 1H, Alkene-CH).

^{13}C NMR ($CDCl_3$, 300 MHz): δ = 47.13, 53.07, 121.40, 125.02, 126.10, 128.55, 129.22, 130.94, 131.77, 132.65, 132.80, 145.02, 147.91, 167.40.

HRMS: m/z Calcd for $C_{19}H_{16}BrN_3O_2$ $[M+H]^+$: 398.0426; found: 398.0435.

(*E*)-Ethyl 3-(4-bromophenyl)-2-((4-(hydroxymethyl)-1*H*-1,2,3-triazol-1-yl)methyl)acrylate (3k)

Yield: 250 mg (78%); white solid; mp 110-112 °C.

^{1}H NMR ($CDCl_3$, 300 MHz): δ = 1.30-1.34 (t, *J* = 6 Hz, 3H, estr-CH_3), 3.09 (s, 1H, CH_2-OH), 4.23-4.31 (q, *J* = 9 Hz, 2H, estr-CH_2), 4.78 (s, 2H, Trz-CH_2), 5.31 (s, 2H, Trz-CH_2-OH), 7.57-7.64 (m, 4H, Aro-H), 7.75 (s, 1H, Trz-CH), 7.97 (s, 1H, Alkene-CH).

^{13}C NMR ($CDCl_3$, 300 MHz): δ = 14.60, 47.06, 56.78, 62.17, 123.42, 124.90, 126.29, 131.65, 132.62, 132.83, 144.69, 147.82, 166.80.

HRMS: m/z Calcd for $C_{15}H_{16}BrN_3O_3$ $[M+H]^+$: 366.0375; found: 366.0383.

(*Z*)-3-(4-Bromophenyl)-2-((4-(hydroxymethyl)-1*H*-1,2,3-triazol-1-yl)methyl)acrylonitrile(3l)

Yield: 274 mg (82%); white solid; mp 114-116 °C.

^{1}H NMR ($CDCl_3$, 300 MHz): δ = 4.65 (s, 2H, Trz-CH_2), 5.17 (s, 2H, Trz-CH_2-OH), 7.28-7.31 (d, *J* = 9 Hz, 2H, Aro-H), 7.39 (s, 1H, Alkene-CH), 7.48-7.51, (d, *J* = 9 Hz, 2H, Aro-H), 7.69 (s, 1H, Trz-CH).

^{13}C NMR ($CDCl_3$, 300 MHz): δ = 48.01, 56.34, 109.87, 118.21, 123.04, 125.43, 131.23, 132.69, 134.36, 147.93, 149.54.

HRMS: m/z Calcd for $C_{13}H_{11}BrN_4O$ $[M+H]^+$: 319.0116; found: 319.0125.

(*E*)-Methyl 2-((4-(hydroxymethyl)-1*H*-1,2,3-triazol-1-yl)methyl)-3-(4-nitrophenyl)acrylate (3m)

Yield: 291 mg (87%); yellow solid; mp 143-145 °C.

^{1}H NMR ($CDCl_3$, 300 MHz): δ = 2.77 (s, 1H, Trz-CH_2OH), 3.86 (s, 3H, estr-CH_3), 4.80 (s, 2H, Trz-CH_2), 5.29 (s, 2H, Trz-CH_2-OH), 7.78 (s, 1H, Trz-CH), 7.90-7.92 (d, *J* = 6 Hz, 2H, Aro-H), 8.06 (s, 1H, Alkene-CH), 8.30-8.33 (d,*J* = 9 Hz, 2H, Aro-H).

^{13}C NMR ($CDCl_3$, 300 MHz): δ = 46.67, 53.16, 56.56, 123.61, 124.34, 128.70, 130.86, 140.31, 143.04, 148.46, 148.60, 166.53.

HRMS: m/z Calcd for $C_{14}H_{14}N_4O_5$ $[M+H]^+$: 319.0964; found: 319.0954.

(*E*)-Methyl 3-(furan-2-yl)-2-((4-phenyl-1*H*-1,2,3-triazol-1-yl)methyl)acrylate (3n)

Yield: 339 mg (80%); reddish oil.

^{1}H NMR ($CDCl_3$, 300 MHz): δ = 3.84 (s, 3H, estr-CH_3), 5.74 (s, 2H, Trz-CH_2), 6.56 (s, 1H, Het-H), 6.98 (s, 1H, Aro-H), 7.30-7.32 (d, *J* = 6Hz, 1H, Het-H), 7.37-7.42 (m, 2H, Aro-H), 7.64 (s, 1H, Trz-CH), 7.73 (s, 1H, Alkene-CH), 7.79-7.82 (m, 2H, Aro-H), 7.84 (s, broad, 1H, Het-H).

^{13}C NMR ($CDCl_3$, 300 MHz): δ = 47.19, 53.03, 113.20, 119.94, 120.31, 126.11, 128.44, 129.15, 130.99, 131.27, 146.86, 150.15, 167.78.

HRMS: m/z Calcd for $C_{17}H_{15}N_3O_3$ $[M+H]^+$: 310.1113; found: 310.1122.

(*E*)-Ethyl 3-(furan-2-yl)-2-((4-phenyl-1*H*-1,2,3-triazol-1-yl)methyl)acrylate (3o)

Yield: 313 mg (76%); yellowish solid; mp 95-97 °C.

^{1}H NMR ($CDCl_3$, 300 MHz): δ = 1.28-1.32 (t, *J* = 6Hz, 3H, estr-CH_3), 4.23-4.29 (q, *J* = 6Hz, 2H, estr-CH_2), 5.72 (s, 2H, Trz-CH_2), 6.54 (s, 1H, Het-H), 6.97 (s, 1H, Aro-H), 7.30-7.39 (m, 3H, Aro-H& Het-H), 7.61 (s, 1H, Trz-CH), 7.71 (s, 1H, Alkene-CH), 7.78-7.81 (m, 2H, Aro-H), 7.85 (s, broad, 1H, Het-H).

^{13}C NMR ($CDCl_3$, 300 MHz): δ = 14.65, 47.11, 61.98, 113.16, 119.71, 120.21, 120.73, 126.02, 128.32, 129.12, 130.90, 131.19, 146.71, 147.75, 150.20, 167.22.

HRMS: m/z Calcd for $C_{18}H_{17}N_3O_3$ $[M+H]^+$: 324.1270; found: 324.1256.

(*E*)-Methyl 2-((4-phenyl-1*H*-1,2,3-triazol-1-yl)methyl)-3-(thiophen-2-yl)acrylate (3p)

Yield: 307 mg (75%); yellowish solid; mp 107-109 °C.

^{1}H NMR ($CDCl_3$, 300 MHz): δ = 3.87 (s, 3H, estr-CH_3), 5.63 (s, 2H, Trz-CH_2), 7.19 (m, 1H, Aro-H), 7.34 (m, 1H, Het-H), 7.41-7.42 (m, 2H, Aro-H), 7.61 (s, 1H, Trz-CH), 7.70 (s, 1H,Alkene-CH), 7.82-7.84 (m, 2H, Aro-H), 7.90 (m, 1H, Het-H), 8.17 (s, 1H, Het-H).

^{13}C NMR ($CDCl_3$, 300 MHz): δ = 30.10, 53.09, 120.23, 128.47, 128.90, 129.16, 129.33, 130.98, 132.11, 134.69, 136.72, 138.28, 148.00, 167.80.

HRMS: m/z Calcd for $C_{17}H_{15}N_3O_2S$ $[M+H]^+$: 326.0885; found: 326.0879.

(*E*)-Ethyl 2-((4-phenyl-1*H*-1,2,3-triazol-1-yl)methyl)-3-(thiophen-2-yl)acrylate (3q)

Yield: 279 mg (70%); yellow oil.

^{1}H NMR ($CDCl_3$, 300 MHz): δ = 1.37-1.41 (t, *J* = 6Hz, 3H, estr-CH_3), 4.35-4.41 (q,*J* = 6Hz, 2H, estr-CH_2), 4.47 (s, 2H, Trz-CH_2), 7.40 (s, 1H, Aro-H), 7.42 (s, 1H, Het-H), 7.46 (s, 1H, Aro-H), 7.48 (s, 1H, Aro-H), 7.50 (s, 1H, Het-H), 7.84 (s, 1H, Trz-CH), 7.86 (s, 1H, Alkene-CH), 7.91-7.93 (m, 1H, Aro-H), 8.01 (s, 2H, Aro-H& Het-H),

^{13}C NMR ($CDCl_3$, 300 MHz): δ = 14.28, 46.98, 61.06, 125.75, 126.14, 126.60, 128.75, 129.00, 131.06, 131.64, 133.13, 134.26, 134.97, 137.70, 147.08, 166.98.

HRMS: m/z Calcd for $C_{18}H_{17}N_3O_2S$ $[M+H]^+$: 340.1041; found: 340.1029.

Spectral data of 3-(bromomethyl)-2*H*-chromen-2-one 4a–c

3-(Bromomethyl)-2*H*-chromen-2-one (4a) [3]

Yield: 186 mg (78%); White crystal; mp 112-114 °C.

^{1}H NMR ($CDCl_3$, 300 MHz): δ = 4.55-4.57 (d, *J* = 6Hz, 2H, CH_2-Br), 7.31-7.57 (m,4H, Aro-H), 7.89-7.91 (d,*J* = 6Hz, 1H, Alkene-CH).

^{13}C NMR ($CDCl_3$, 300 MHz): δ = 41.49, 117.02, 119.16, 125.14, 125.33, 128.50, 132.45, 141.62, 153.90, 160.47.

HRMS: m/z Calcd for $C_{10}H_7BrO_2$ $[M+H]^+$: 238.9629; found: 238.9618.

3-(Bromomethyl)-8-methoxy-2*H*-chromen-2-one (4b)

Yield: 208 mg (87%); White crystals; mp 151-153 °C.

^{1}H NMR (DMSO-D_6, 300 MHz): δ = 3.92 (s, 3H, Aro-O-CH_3), 4.63 (s, 2H, CH_2-Br), 7.31-7.32 (m, 3H, Aro-H), 8.22 (s, 1H, Alkene-CH).

^{13}C NMR (DMSO-D_6, 300 MHz): δ = 42.02, 56.61, 115.10, 119.63, 120.28, 125.22, 125.26, 143.01, 143.23, 146.88, 159.50.

HRMS: m/z Calcd for $C_{11}H_9BrO_3$ $[M+H]^+$: 267.9735; found: 267.9724.

6-Bromo-3-(bromomethyl)-2*H*-chromen-2-one (4c)

Yield: 181 mg (75%); White crystals; mp 125-127 °C.

^{1}H NMR (CDCl3, 300 MHz,): δ = 4.42 (s, 2H, CH_2-Br), 7.23-7.25 (d, *J* = 6 Hz, 1H, Aro-H), 7.61-7.62 (d, *J* = 3Hz, 1H, Aro-H), 7.65 (s, 1H, Aro-H), 7.79 (s, 1H, Alkene-CH).

^{13}C NMR (CDCl3, 300 MHz,): δ = 27.14, 117.31, 118.47, 120.40, 126.79, 130.29, 134.87, 140.49, 152.53, 159.22.

HRMS: m/z Calcd for $C_{10}H_6Br_2O_2$ $[M+H]^+$: 317.8714; found: 317.8703.

References

1. Chandrasekhar, S.; Basu, D.; Rambabu, Ch. *Tetrahedron Lett*. **2006**, *47*, 3059. doi:10.1016/j.tetlet.2006.03.037
2. Sreedhar, B.; Reddy, P. S.; Kumar, N. S. *Tetrahedron Lett*. **2006**, *47*, 3055. doi:10.1016/j.tetlet.2006.03.007
3. Tan, Y.; Yang, X. –D.; Liu, W. –J.; Sun, X. –W. *Tetrahedron Lett.* **2014**, *55,* 6105-6108 . doi:10.1016/j.tetlet.2014.09.045
